# Youth Experiences With Referrals to Mental Health Services in Canada: Protocol for a Web-Based Cross-Sectional Survey Study

**DOI:** 10.2196/16945

**Published:** 2020-03-24

**Authors:** Shalini Lal, Danielle Joanna Starcevic, Rebecca Fuhrer

**Affiliations:** 1 School of Rehabilitation, Faculty of Medicine University of Montréal Montréal, QC Canada; 2 Youth Mental Health and Technology Lab University of Montréal Hospital Research Centre Montréal, QC Canada; 3 ACCESS Open Minds (Pan-Canadian Youth Mental Health Services Research Network) Douglas Mental Health University Institute Montréal, QC Canada; 4 Prevention and Early Intervention Program for Psychosis (PEPP) Douglas Mental Health University Institute Montréal, QC Canada; 5 Dept of Epidemiology, Biostatistics and Occupational Health Faculty of Medicine McGill University Montréal, QC Canada

**Keywords:** mental disorders, health care quality, access, and evaluation, mental health, psychology, telemedicine, young adult, health services accessibility, technology, referral and consultation

## Abstract

**Background:**

Youth mental health is an important public health concern affecting low-, middle-, and high-income countries, and many young people in need of mental health services do not receive the care they need when they need it. An early step in accessing mental health care is the referral process, yet most of the research done on pathways to care has focused on clinical populations (eg, first-episode psychosis) recruited from mental health care settings. There has been limited research attention on the experiences of referral to mental health services from the perspectives of youth recruited from the general population who may or may not have received the services they need.

**Objective:**

This study aims to investigate the experiences that youth between the ages of 17 and 30 years have with referrals to mental health services and to better understand their perspectives on the use of technology to facilitate referrals.

**Methods:**

This study will use a cross-sectional, Web-based survey design. A convenience sample of 400 participants from 3 Canadian provinces (Quebec, Ontario, and British Columbia), between the ages of 17 and 30 years, will be recruited via Facebook and will be invited to complete a Web-based survey anonymously. A questionnaire including a series of quantitative and qualitative questions will ask participants about their sociodemographic characteristics, past experiences with referral and access to mental health services, and opinions about using technology to facilitate the referral process.

**Results:**

Participant recruitment is planned to be initiated by early January 2020 and is estimated to be completed by May 2020. Data will be analyzed using descriptive statistics and logistic regression or chi-square tests for quantitative data, and descriptive content analysis will be used for the qualitative data.

**Conclusions:**

The results of this study can help inform the improvement of referral policies and procedures in youth mental health service delivery. A better understanding of young people’s perspectives on referral processes and their opinions on how these processes can be improved are essential to providing appropriate and timely access to mental health care.

**International Registered Report Identifier (IRRID):**

PRR1-10.2196/16945

## Introduction

### Background

Mental health disorders are among the leading causes of morbidity and mortality in young people aged between 10 and 24 years and are major contributors to the global burden of disease [1]. Most mental health disorders will emerge by the age of 24 years [2], and one in every four to five individuals aged 12 to 24 years will experience at least one mental illness in any given year [3]. The prevalence of mental health disorders in young people combined with poor access to timely and appropriate care is an issue affecting low-, middle-, and high-income countries [4]. For example, in Canada, research has shown that youth aged 15 to 24 years have higher rates of mental illness than any other age group, with approximately 8% of youth experiencing a mood disorder and approximately 12% experiencing a substance use disorder [5]. Despite such high rates of mental illness, a Canadian Community Health Survey showed that only 12% of Canadians aged between 15 and 24 years reported consulting mental health professionals about emotional, mental, or substance use problems within the previous 12 months [6]. In addition, only about half of youth living with a mental health disorder consulted professional services (eg, psychologists, psychiatrists, nurses, and social workers) within the last 12 months, indicating the possibility of unmet mental health needs among young Canadians [6]. Moreover, the pathways for those who attempt to seek help but do not receive it are unclear.

### Accessing Mental Health Services

The barriers that youth face when trying to access mental health services relate to the individual, the society, and the mental health care system. Common help-seeking barriers reported in the literature include stigma, lack of confidentiality and trust, lack of knowledge regarding symptoms and service options, and self-reliance [7-15]. Even when the help-seeking process has been initiated by a young person and/or their caregivers, there can be several challenges in accessing services [7], such as long wait lists. In a Canadian survey of agencies providing youth mental health care 91.3% (106/116) of agencies reported a waiting list for one or more of the services they offered [16]. Only 63.7% (65/102) of these agencies were mostly or always able to meet the Canadian Psychiatric Association wait time benchmarks for provision of urgent care within 2 weeks, and only 31.4% (32/102) were mostly or always able to meet the 1-month benchmark for scheduled care [16].

According to the Fraser Institute’s 2018 national waiting list survey conducted in Canada, the median wait time from referral by a general practitioner to a psychiatrist is 20.8 weeks, which is slightly higher than the weighted median wait time of 19.8 weeks across medical specialties and higher than the wait time for other specialties such as general surgery (12.9 weeks), nonurgent (elective) cardiovascular surgery (9.9 weeks), internal medicine (13.3 weeks), radiation oncology (4.0 weeks), and medical oncology (3.8 weeks) [17]. It should also be noted that response rates for psychiatric wait times (186/4099, 4.54%) were much lower compared with response rates for wait times for other medical services (1718/10,209, 16.82%), and there is, therefore, a potential for bias within the results because of the low response rates for psychiatric services [17].

Certain demographic factors, such as gender, race and ethnicity, housing stability, immigration status, employment and education status, and location, have also been noted in the literature to be associated with access to mental health care. For example, barriers faced by rural youth are a particular concern in Canada; about 10 million or one-third of Canadians live in rural communities [18]. Parents of youth with mental illnesses living in rural areas in Canada have highlighted the lack of resources and services, funding issues, long waiting lists, and distance to services as major barriers to accessing appropriate mental health care for their children [19].

In addition, research has shown that immigrants, refugees, and members of visible minority and ethnocultural groups have limited access to mental health services. For example, a 2007 survey of the catchment area of a comprehensive community clinic located in Montréal found that ethnocultural minority immigrant groups born in the Caribbean, Vietnam, or the Philippines were significantly less likely than their Canadian-born peers to use mental health services, despite using medical services for physical health issues at similar rates [20]. Importantly, the lower rates of mental health service use among immigrant groups could not be attributed to other sociodemographic differences (ie, differences between groups in sex, age, marital status, employment status, and citizenship status), differences in physical or psychological symptoms or distress, length of stay in Canada, or use of alternative resources [20]. Limited knowledge about symptoms of mental illness and the services available, cultural and language barriers, and discrimination are some examples of obstacles faced by immigrants who require mental health care [21]. The barriers that young people, especially those in certain demographic groups, experience when seeking help for mental health concerns can influence their pathways to care and the delays associated with this process.

Pathways to care are defined by the help-seeking behavior of the individual, the sequence of contacts the individual has with mental health services, and how those services respond to the needs of the individual, for example, through referrals to appropriate mental health care services [22,23]. For youth, getting a professional referral to appropriate mental health services can be a harrowing process. For example, within the studies that reported total contacts before receiving specific health services, the number of contacts ranged from 0 to 15 contacts (with a pooled mean of 2.9 contacts) per participant and included medical and nonmedical professionals, friends and family, health care institutions, the justice system, traditional healers, and electronic mental (e-mental) health contacts [24]. The mean duration of untreated psychosis (DUP) for youth experiencing first-episode psychosis ranged from 1.5 to 102 weeks (with the median ranging from 8 to 70 weeks) across 23 studies [24]. Duration of untreated illness (DUI) for youth experiencing a variety of mental health problems ranged from 1 week to 45 years across 15 studies (pooled mean or median were not specified in this review) [24]. Importantly, among the studies that differentiated between referral delays and help seeking delays, the majority reported that referral delays were longer than delays related to help seeking behavior within both DUP and DUI [24].

Various solutions have been implemented to improve pathways to mental health care, for example, the use of open referral systems that provide access to services through any sources of referral, including the individual themselves, family, or other sources [25]. However, the use of open referral systems in youth mental health care have traditionally relied on paper-based methods with limited technology-enabled systems in place to triage, track, and monitor referrals over time. For example, in Canada, the Prevention and Early Intervention Psychosis Program-Montréal offers an open referral approach in which anyone (eg, the individual experiencing mental health–related issues, their families, teachers, and emergency departments) can contact the clinic in person, by phone, or by email [26,27]. However, despite this approach, most referrals to the clinic still come from formal health services [27], indicating that barriers to self-referral exist within open referral systems that use traditional methods of referral (eg, phone, paper, and fax).

More recently, information and communication technologies have been leveraged to improve the efficiency of the referral process to mental health services. Kim et al [28] evaluated the implementation of a Web-based self-referral system in a mental health clinic for adults. In this study, 30% of new adult patients to the clinic were introduced by the Web-based self-referral tool; 80% (45/56) of the clients were satisfied with the Web-based tool, 93% (53/57) said the response to their self-referral was timely, 89% (50/56) felt comfortable using the system, 51% (29/57) said the system improved the quality of health care received, and 89% (50/56) would use a similar Web-based referral system if available [28]. After the trial, the tool was successfully adopted by the clinic [28]. In another recent study, the use of an electronic referral form connected to an electronic health record was implemented to facilitate care coordination among a multidisciplinary team providing mental health services to youth [29]. However, the implementation of Web-based referral services that directly connect youth to public mental health care services is still limited, and to the authors’ knowledge, no studies have focused on youth’s perspectives in relation to the potential use of technology to complete a self-referral process (eg, perceived benefits, concerns, and suggestions). Such knowledge is important to ascertain, given that research has shown that although youth are open to the idea of using technology in the context of mental health care [30-34], there is variation in terms of the types of Web-based services to which they are receptive [32,33].

In addition, although previous studies have explored the pathways that youth take to access mental health services, the majority of this research has either focused on pathways pertaining to specific mental health concerns (eg, first-episode psychosis), has only recruited clinical populations from within mental health care settings, or has not reported the referral sources within these pathways [24]. Regarding the latter, only 22 of the 45 pathways to care studies included in the review by Macdonald et al [24] examined and reported on referral sources. The referral pathways of youth in the general population who do not successfully access services have been largely ignored. In addition, although several studies have reported on the length of time between the first contact and the reception of mental health services [35-45], little attention has been paid to the actual process of referral. Moreover, further research is needed to have a more complete understanding of the experiences that youth in the general population have with the process of referral to mental health care.

### This Study

This study investigates the experiences that Canadian youth between the ages of 17 and 30 years have with being referred to mental health services. The study will examine the following subquestions: (1) What mental health concerns do youth report seeking services for in Canada? (2) What are the pathways and processes that youth experience when trying to access mental health services? (3) What are the barriers and obstacles that youth face in the process of referral to mental health services? (4) What, if any, sociodemographic factors (eg, age and gender) are associated with access to mental health services and mental health service referrals? and (5) What are the views of youth on the use of technology to facilitate self-referrals to mental health services?

## Methods

### Design

This study will be conducted using a cross-sectional research design. The main method of data collection will be an anonymous Web-based survey that includes open and closed questions. The ability to gather precise numerical data, study large populations, and have generalizability are all important benefits of collecting quantitative data [46]. However, the experiences that youth have with accessing and being referred to mental health care services vary largely, and the restricted nature of quantitative questions may lead to important information being overlooked. Thus, we will also collect qualitative data in conjunction with the quantitative methods to allow for a deeper understanding of young people’s referral experiences [46].

### Study Population and Inclusion and Exclusion Criteria

To be included in the study, participants must be between the ages of 17 and 30 years; have reported accessing or trying to access mental health services in Quebec, Ontario, or British Columbia within the past 5 years; be able to complete the survey in English or French; and give online consent to participate in the study. Participants will be excluded from the study if they do not provide online consent for participation or if the participant does not click on the submit button at the end of the survey.

The definition of youth varies between contexts but commonly includes the period between the ages of 12 and 25 years [47,48]. This study will focus on interviewing individuals aged between 17 and 30 years to ensure that the experiences reported in the survey occurred when participants were within the age range (ie, 12-25 years) considered youth.

### Sampling, Sample Size, Recruitment, and Endpoint

#### Sampling Method

A convenience sampling method will be used for this study. Convenience sampling involves sampling participants who are available to approach at the time of recruitment, for example, recruiting participants from a specific university course or club [49]. It is a useful method to recruit broad target populations and has the additional advantages of being inexpensive and time efficient [49]. Previous research has used convenience sampling with Web-based surveys to better understand youth experiences with mental health care or mental health, specifically within the context of e-mental health [13,34,50], including a recent Canadian study conducted using a Web-based survey with youth aged 17 to 24 years recruited from the general population on their experiences with Web-based and traditional mental health resources [33].

### Sample Size and Endpoint

The estimated sample size is 400. This was estimated using Cochran’s sample size formula for a single population proportion for large population using a 95% CI, a margin of error of 5%, and an estimate of the sample proportion of 50% [51,52]. The CI and margin of error were selected based on standard values used for research. The sample proportion is an estimate of the proportion of the population that has an attribute we want to measure. A sample proportion of 50% was chosen because this value provides the largest sample size estimate [51-53]. A sample size of 400 will also allow us to compare reported use and satisfaction with mental health services between demographic groups. For example, if we wanted to detect a 20% difference in reported satisfaction (satisfied vs unsatisfied) between females and males using a power of 80% and a margin of error of 0.05, we would need to have about 80 participants in each gender group. Furthermore, we plan to perform regression analyses to explore any potential associations between 12 sociodemographic variables and reported use and satisfaction with mental health services. Given the rule of thumb N>104+m, in which N is the number of participants and m is the number of variables in the regression, our sample size of 400 should be sufficient for such analyses [54]. The end point of the study will occur when the desired sample size of 400 is reached. On the basis of previous studies using similar methodologies and recruitment methods, this is estimated to take approximately 4 months [13,33], but the timeline will be adjusted to reflect the actual rate of recruitment.

#### Recruitment

Participants will be recruited through Facebook advertisements on pages targeted toward youth living in Quebec, Ontario, and British Columbia between the ages of 17 and 30 years. This includes the Facebook pages of relevant groups or organizations on university, high school, and Collège d’enseignement général et professionnel (CEGEP) campuses; community mental health organizations; and general Facebook pages accessed by youth. We will initially target university- and community-based Facebook pages that the research team has access to. To maximize the representativeness of our sample population, we will also randomly select universities, high schools, CEGEPs, and youth-focused organizations from comprehensive provincial lists of these institutions and identify whether the selected institutions have Facebook pages where we could potentially post our flyer. We will contact the selected Facebook pages of these groups via private message on Facebook and invite them to post the study advertisement with the link to the survey on their pages.

Facebook recruitment was chosen because of the large proportion of Canadian youth who report using Facebook. For example, a recent Web-based survey study of 1500 participants showed that 95% of Canadians between the ages of 18 and 24 years and 94% of Canadians between the ages of 24 and 34 years have a Facebook account, with 88% and 82% being monthly active users, respectively [55]. Facebook use is high (above 75%) across income levels, education levels, and employment levels [55]. In addition, previous studies have found that participants recruited through Facebook were representative of the populations from which they were sampled [56-58]. Notably, Fenner et al [56] reported that the proportions of foreign-born and indigenous participants recruited through Facebook were similar to the proportions of foreign-born and indigenous individuals in the target population (46/276, 16.7% vs 19.8% were foreign born and 3/278, 1.1% vs 0.9% were indigenous in the study and target populations, respectively). Importantly, in our study, to conserve the anonymity of participants, Facebook accounts will not be linked to the questionnaire.

### Questionnaire Development

The questionnaire was developed using the Research and Electronic Data Capture (REDCap) (Vanderbilt University) tool hosted internally at the Centre de Recherche du Centre Hospitalier de l’Université de Montréal (CRCHUM), which will also be used to deploy and manage the questionnaire. REDCap is a secure application that provides an interface for data entry, audit trails for tracking data export and analysis, automated data export to various statistical software, and the ability to import data from outside sources [59]. All data will be collected anonymously using the REDCap tool, and answers will not be attributed to any individual.

The questionnaire was developed based on a previous, brief consultation questionnaire that the principal investigator (PI) had developed on the subject of youth experiences of being referred to mental health services in Canada and their point of view on the use of a Web-based self-referral tool for mental health services before obtaining funds for this project. Building on this consultation questionnaire, the current version of the questionnaire was further developed through a literature review and discussion between the research team (research assistant, PI, and a research coordinator in the PI’s laboratory). Following this, a more systematic process was used to elicit feedback from 5 reviewers, including research staff and student interns, of whom 3 were individuals with disclosed lived experience accessing mental health care in Canada. Each reviewer provided individual feedback using a standardized feedback form that was used to revise the questionnaire.

The questions, introduction, and consent form subsequently underwent an extensive translation process from English to French, including forward and back translation involving 4 bilingual members of the research team, of whom 2 are native French speakers and 2 are native English speakers. The questionnaire was then piloted in English and in French with 2 additional members from the PI’s laboratory (who are also individuals within the target age range of the population being recruited), and feedback was obtained to enhance readability and the content of the questionnaire, before being finalized. During this pilot study, the questionnaire took between 10 and 15 min, on average, to complete.

#### Description of the Questionnaire

The questionnaire collects data on demographic information, past experiences accessing mental health service referrals in Canada, and opinions on using technology to facilitate referral to mental health services (see Multimedia Appendices 1 and 2). The questionnaire includes a total of 51 questions; 8 questions are open ended and 43 are closed questions (including 4 with Likert attribute responses and 39 with multiple choice options). To ease the burden of participation, the questionnaire includes skip logic to ensure participants only answer questions that are relevant to their experiences. The questionnaire includes the following structure: screening questions, consent form, demographic questions, past experiences with mental health referrals, and views on a Web-based self-referral tool described in further detail below.

#### Screening Questions

**Figure 1:**
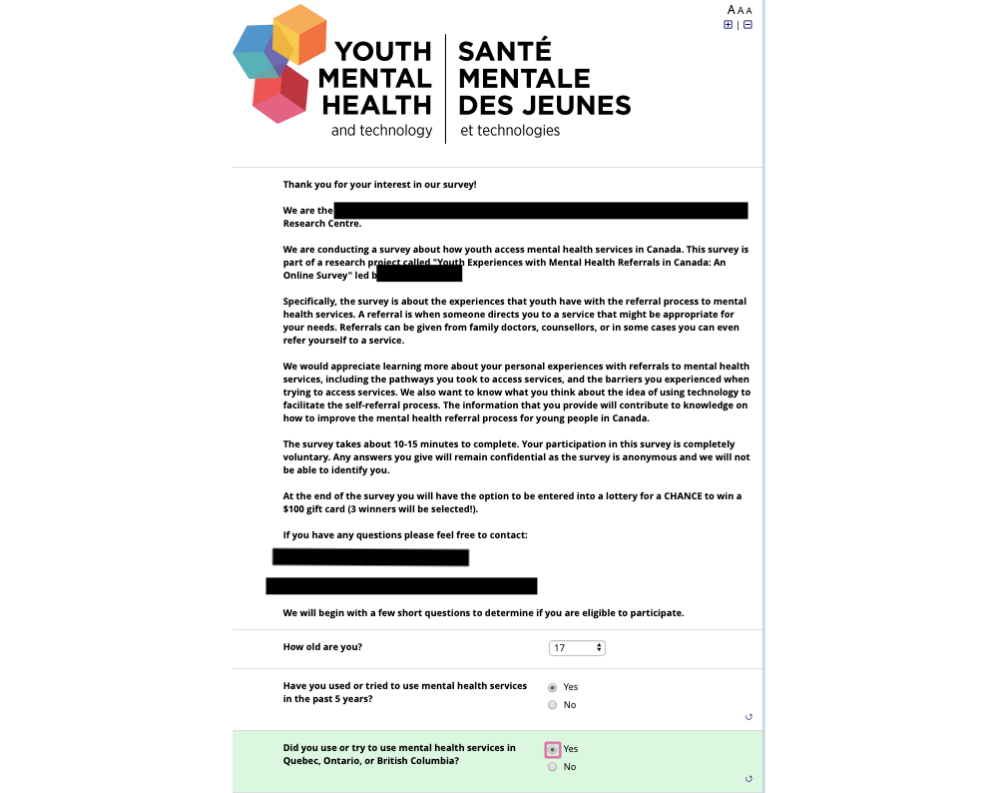
Questionnaire introduction and screening questions.

The questionnaire includes 3 screening questions to assess participants’ eligibility to participate in the study. The questions ask about age and whether the individual has accessed or tried to access mental health services in Ontario, Quebec, or British Columbia (see Figure 1*).*

#### Consent Form

Once participants are deemed eligible to participate, a consent form appears that explains the purpose of the study, what the data will be used for, how the data will be stored, requirements of the participant, and any potential benefits or risks incurred by participating, and that consent for participation can be withdrawn at any point before submission of the questionnaire. Owing to the anonymous nature of the questionnaire, participant information cannot be withdrawn after a participant submits their data, as the researchers will not know which data belong to each participant. Participants are informed of this in the consent form.

#### Demographic Questions

A total of 15 demographic questions (3 open ended and 12 close ended) are included in the questionnaire. A series of 7 questions ask participants about their age, gender, level of education, transgender status, immigration status, and ethnicity. Age is asked at the beginning of the questionnaire to determine eligibility to participate in the study. Owing to the complex nature of the topic, the question about ethnicity is a close-ended question followed by an open-ended option that invites participants to self-report their ethnic identity. A series of 7 questions ask participants about their age, location, vocational status, living situation, annual household income, and length of stay in Canada (if not born in Canada). These questions are asked in relation to their status when participants first accessed or tried to access mental health services in the past 5 years.

#### Past Experiences With Mental Health Referrals

A total of 28 questions (3 open ended, 3 Likert, and 22 close ended) ask participants about past experiences accessing mental health services and referrals. Questions ask about the first-time participants accessed or tried to access mental health services in the past 5 years, including the type of mental health concern that prompted help seeking, the first person they approached for help, and whether mental health services were actually received for the concern or not. If participants received mental health services they are then asked for information about their experiences with the first mental health service provider they contacted, including questions about the setting of the service, the role of the professional contacted (ie, doctor and psychologist), the type of service received, who recommended the first contact to the participant, the steps the participant took to access the first contact, the method of contact (including forms), wait times, obstacles, satisfaction with the referral process, and whether an additional referral to a second mental health service provider (second contact) was provided. If a referral to a second contact was provided, participants are then asked for similar information as above, up to a maximum of 2 contacts. Finally, participants will be asked for information about their overall pathway to receiving appropriate mental health services, including number of contacts, length of time, and overall satisfaction. Participants who sought help but did not actually receive mental health services will only be asked about the obstacles that kept them from receiving mental health services.

#### Perspectives on Using Technology to Facilitate Referral to Mental Health Services

The last section of the questionnaire includes a series of 4 questions (3 open ended and 1 Likert) that ask participants about their perspectives on using technology to facilitate referral to mental health services. Questions cover topics such as receptivity to using technology to facilitate referral, including potential benefits and concerns and recommendations related to this approach.

### Follow-Up and Compensation

#### Collection of Personal Information

After completing the questionnaire, participants will have the option to provide their contact information, including their first name, email address, and phone number. If participants choose to provide contact information, it will be used to update participants on the results of this study and invite them to future studies and activities. Participants will also have the option to be entered into a lottery to win one of three Can $100 (US $74.96) gift cards on completion of the questionnaire. Participants do not have to agree to receive updates or agree to be invited to participate in future studies to enter the gift card draw. Contact information will be collected using a separate form, so any personal information cannot be linked to the data provided in the Web-based questionnaire.

#### Concern for Welfare

Mental health can be a sensitive topic, and our questionnaire has the potential to trigger emotional reactions. In the informed consent section of the questionnaire, we will mitigate this risk by warning participants that the questions might be of a sensitive nature, and participants will be assured that they can skip any question that they do not feel comfortable answering. We will also provide resources for free online supports, and a list of resources will be offered at the end of the questionnaire for any participant who may feel the need to speak with someone on completion of the questionnaire.

#### Data Storage

All data will be stored on secure internal servers at the CRCHUM and will be directly downloaded from the REDCap tool and stored on a password-protected computer in a locked room at the CRCHUM. Data will be stored for 10 years at the CRCHUM before they are destroyed. The 10 principles outlined in the Personal Information Protection and Electronics Document Act were considered throughout the development of the methodology to ensure the proper handling of the personal information collected in the study [60].

## Results

### Current Progress

This study has been approved by the ethics review board at the University of Montréal Hospital Research Centre (project #18.255) and additionally received approval from the ethics review board at McGill University (project A04-B19-19B). The recruitment and data collection phases are estimated to be initiated in January 2020 and to be completed by May 2020.

### Data Analysis Plan

Data will be analyzed and summarized in a final report of the findings but will remain anonymous and nonidentifiable. Quantitative data will be analyzed using R statistical software (Lucent Technologies, Murray Hill, NJ). R is a free statistical computing language developed at Lucent Technologies (formerly AT&T and Bell laboratories) [61]. Simple descriptive statistics (eg, mean, standard deviation, and frequencies) will be used to analyze demographic data and data from close-ended and Likert questions. For example, we will report on the number and percentage of the total participants who identify their gender as each of the following: *Male*, *Female*, *Non-Binary*, *Other*, and *Prefer not to say*. Open-ended questions will be analyzed using descriptive content analysis. Specifically, a manifest analysis methodology with an inductive coding system will be used to identify and categorize qualitative data from open-ended questions [62]. In manifest analyses, researchers describe reported answers, often staying close to and using words given in the original text [62]. A manifest analysis was chosen because of the anonymous nature of the questionnaire, as it would be difficult for the researchers to interpret and ascribe meaning to the given answers without certain contextual clues that might be present in other qualitative data collection methods (eg, vocal tone in interviews or focus groups). Using inductive coding, we will create codes for the categorization of data during analysis, as opposed to using predetermined codes [62]. The flexible and data-driven nature of inductive coding is preferable, considering the heterogeneity of the survey sample and the potential for unanticipated themes to arise in the data. At least three members of the research team will be involved in the content analysis process. We also plan to quantify key topics identified by participants, for example, in the form of frequencies expressed as percentages. Themes identified in the descriptive content analysis will be used to supplement and support quantitative data regarding young people’s experiences of referral pathways and views on the use of Web-based technology to facilitate referrals. Logistic regression or chi-square tests will be used to explore whether any sociodemographic differences exist in mental health service use, and satisfaction with mental health care services depending on the nature of the data collected. For example, we will analyze whether there are any significant differences between genders (*P*<.05) in the number of participants that indicate they accessed mental health care services for their concerns.

## Discussion

### Principal Findings

The results of this survey will help expand knowledge about the accessibility of mental health services in Canada. Specifically, the results will provide insights into the referral processes and pathways experienced by youth, including those who may not yet have received appropriate assessment and care. This study will build on previous pathways to youth mental health services research that has primarily focused on youth experiencing first episode psychosis [10,24,35,36,63-68]. Our results will help create a more comprehensive understanding of the mental health referral experiences of young Canadians.

In addition, although previous research has indicated that youth who self-refer are likely to meet diagnostic thresholds for mental illnesses [69] and that electronic referral systems can increase the use of appropriate mental health services in youth-centered settings [29], limited research exists on the perspectives of youth in using technology to facilitate the process of referral to mental health services. Their views on this topic are important to ascertain, especially given that research has shown that the level of receptivity young people have with using technology in relation to mental health care varies depending on the type of service that is being proposed to be delivered via technology [32,33]. In addition, studies show that youth have reported hesitations or challenges with Web-based mental health services, including limited knowledge about internet search strategies, concerns about the way information is presented online (particularly the validity or quality of information and comprehension of the information), lack of interest, lack of time, cost of internet access, the need for more information on e-mental health services, and concerns about privacy online [31,70]. However, these studies address e-mental health services more generally or cover other types of e-mental health interventions, and thus, there remains a need for research on youth concerns specific to the use of technology to facilitate referrals.

### Implications for Improving Services

The knowledge gained from this study will help inform the improvement of the referral process for Canadian mental health services. Information collected regarding the mental health concerns that youth report seeking help for, the pathways and processes that youth experience when seeking help, and the barriers that youth commonly face during the referral process can be used by mental health services to help improve their accessibility for young people. In addition, if the results of this study reveal important sociodemographic differences in satisfaction with referral pathways and access to appropriate services, such information can be used by mental health services and Canadian public health agencies to prioritize addressing such determinants to reduce the barriers that certain youth may face in accessing mental health care. If youth further indicate that they are open to and likely to use a Web-based system to facilitate referrals to mental health services, health services and programs can work toward incorporating technology in their referral process.

### Implications for Future Research

Although this study includes some qualitative questions about the mental health referral experience for youth, more in-depth opportunities to explore the topic of referral may be warranted through interviews and focus groups. In particular, if this study identifies common themes among answers to qualitative questions after analysis, these themes may be identified as essential topics to explore in greater detail through qualitative methodology.

### Strengths and Limitations

This study benefits from the data collection instrument, participant eligibility criteria, and recruitment method. We chose a Web-based survey design because of the benefits that this survey method offers, especially for data collection on sensitive and stigmatized topics such as mental health [71]. Owing to the high levels of stigma that surround mental illness, some individuals may be more comfortable sharing their experiences using an anonymous tool such as a Web-based questionnaire [14,71,72]. Confidentiality and privacy are always concerns in an online environment, especially for sensitive information such as personal mental health experiences [73]. The anonymous nature of the Web-based questionnaire instrument minimizes the risks related to privacy and confidentiality for participants. Although there is always the possibility of interference from third parties in online environments, hosting the questionnaire on a secure server minimizes the risk of such interference.

In addition, past studies related to pathways to mental health care have primarily sampled from clinical populations who have successfully accessed services [24]. Our inclusive eligibility criteria ensure that we will be able to gather information from individuals who do and do not successfully access mental health services to better understand the experiences and obstacles faced by a broader range of youth. Finally, given the ubiquitous nature of Facebook use among young people [55], recruiting participants via Facebook allows us to access portions of the target population who we might not be able to access using other recruitment methods such as email listservs. Importantly, many of the targeted recruitment pages on Facebook (ie, university, high school, and CEGEP groups; community mental health organizations; and general youth-oriented pages) are publicly available, meaning individuals do not need to have personal Facebook accounts to access the posts on these pages. The public nature of these pages additionally increases our ability to access hard to reach portions of the target population.

There are various limitations to our study. Sampling bias is a concern with using convenience sampling methods. It is possible that the sample we collect will be systematically different than the general population of Canadian youth, which may cause our study to be biased and will limit the generalizability of our results. We will monitor this throughout the recruitment process and adjust our study advertising strategy accordingly. For example, given that immigrants, refugees, and members of visible minority and ethnocultural groups often face accessibility issues when it comes to mental health services, we want to ensure that we are appropriately sampling from these groups. Previous research has found that targeted Facebook advertisements have been successful in recruiting significantly greater proportions of foreign-born participants than general advertisements [59]. Therefore, we will target recruitment through Facebook pages that are geared toward these groups (eg, Facebook pages intended for international students on university campuses and cultural groups). Furthermore, we will only be sampling from 3 provinces in Canada. As health care is managed at the provincial level, there might be some important differences in the pathways to mental health care between provinces. The results from our study, therefore, might not reflect the experiences and perspectives of youth living in other provinces in the country. In addition, social desirability bias, in which participants answer questions in a way that they believe are socially acceptable, rather than telling the truth, is also a concern in survey studies and could limit the validity of our results [74]. Low response rates are another common concern with Web-based survey studies, and there is the potential that we will have slow or low response rates for our questionnaire. We will mitigate this possibility by increasing our selection of Facebook pages over time or by using alternative advertisement strategies (eg, paid Facebook advertisements) to meet our desired sample size.

### Conclusions

Overall, this study will contribute to the knowledge of youth experiences of referrals to mental health services in Canada. Our results will expand on previous studies of pathways to care and can be used to inform the improvement of referral policies and procedures in youth mental health service delivery. A better understanding of young people’s perspectives on referral processes and their opinions on how these processes can be improved are essential to providing appropriate and timely access to mental health care.

## Appendix

Multimedia Appendix 1 Web-based questionnaire samples.

Multimedia Appendix 2 Questionnaire.

Multimedia Appendix 3 Peer review reports from Mitacs Accelerate.

## Abbreviations

CEGEP: Collège d'enseignement général et professionnel
CRCHUM: Centre de Recherche du Centre Hospitalier de l’Université de Montréal
DUI: duration of untreated illness
DUP: duration of untreated psychosis
e-mental: electronic mental
PI: principal investigator
REDCap: Research and Electronic Data Capture
