# Correction: Youth Experiences With Referrals to Mental Health Services in Canada: Protocol for a Web-Based Cross-Sectional Survey Study

**DOI:** 10.2196/19019

**Published:** 2020-06-12

**Authors:** Shalini Lal, Danielle Joanna Starcevic, Rebecca Fuhrer

**Affiliations:** 1 School of Rehabilitation, Faculty of Medicine University of Montréal Montréal, QC Canada; 2 Youth Mental Health and Technology Lab University of Montréal Hospital Research Centre Montréal, QC Canada; 3 ACCESS Open Minds (Pan-Canadian Youth Mental Health Services Research Network) Douglas Mental Health University Institute Montréal, QC Canada; 4 Prevention and Early Intervention Program for Psychosis (PEPP) Douglas Mental Health University Institute Montréal, QC Canada; 5 Dept of Epidemiology, Biostatistics and Occupational Health Faculty of Medicine McGill University Montréal, QC Canada

In “Youth Experiences With Referrals to Mental Health Services in Canada: Protocol for a Web-Based Cross-Sectional Survey Study” (JMIR Res Protoc 2020;9(3):e16945) the authors noted a footnote was erroneously omitted from the text of Multimedia Appendix 2. The footnote has been added to Multimedia Appendix 2, and is positioned under the question “What are the ethnic or cultural origins of your ancestors? [Please check all that apply]”. The footnote reads:

Ethnicity question and responses were adapted using information from the following documents: 1) Statistics Canada Census of Population 2016, Appendix 5.1 Ethnic Origins disseminated from 2016, 2011, and 2006. Retrieved from: https://www12.statcan.gc.ca/census-recensement/2016/ref/dict/app-ann/a5_1-eng.cfm, 2) Archived Census 2A-L - 2016. Retrieved from: http://www23.statcan.gc.ca/imdb/p3Instr.pl?Function=getInstrumentList& Item_Id=295122&UL=1V&, 3) Ethnic Origin Reference Guide, Census of Population, 2016. Retrieved from: https://www12.statcan.gc.ca/census-recensement/2016/ref/guides/008/98-500-x2016008-eng.cfm & 4) Data Tables 2016 Census: Ethnic Origins. Retrieved from: https://www12.statcan.gc.ca/census-recensement/2016/dp-pd/dt-td/Av-eng.cfm?LANG=E& APATH=3&DETAIL=0&DIM=1&FL=A&FREE=0& GC=0&GID=0&GK=0&GRP=1&PID=112450& PRID=10&PTYPE=109445&S=0& SHOWALL=0&SUB=0&Temporal=2017& THEME=120&VID=29591&VNAMEE=&VNAMEF=

The corrected Multimedia Appendix 2 file will be uploaded to the online version of the original paper on the JMIR website on June 12, 2020, together with the publication of this correction notice. Because this was made after submission to PubMed, PubMed Central, and other full-text repositories, the corrected article has also been resubmitted to those repositories.

## Appendix

Multimedia Appendix 2 Questionnaire.

