# Supplementary material for: Youth Experiences With Referrals to Mental Health Services in Canada: Protocol for a Web-Based Cross-Sectional Survey Study
Source: JMIR Res Protoc. 2020 Mar 24;9(3):e16945. doi: 10.2196/16945 (PMC7139421; doi:10.2196/16945)

Appendix A: Web-based questionnaire samples

#

Introduction & Eligibility
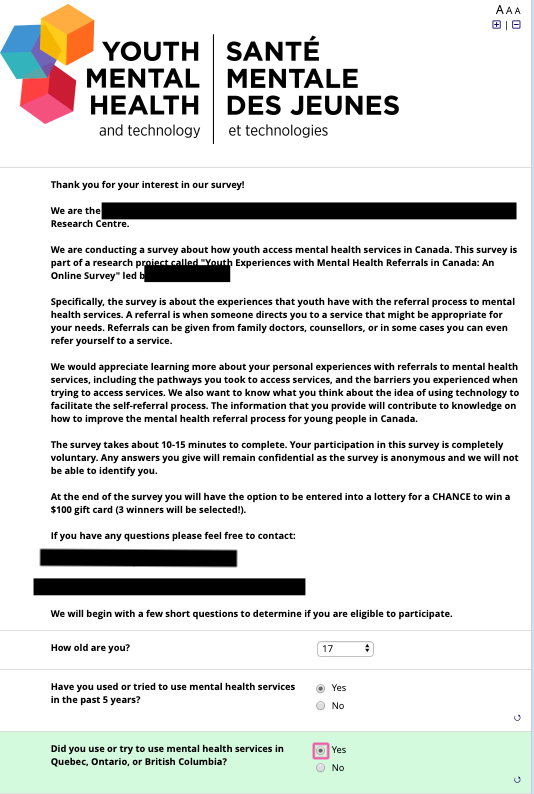


Demographic Questions


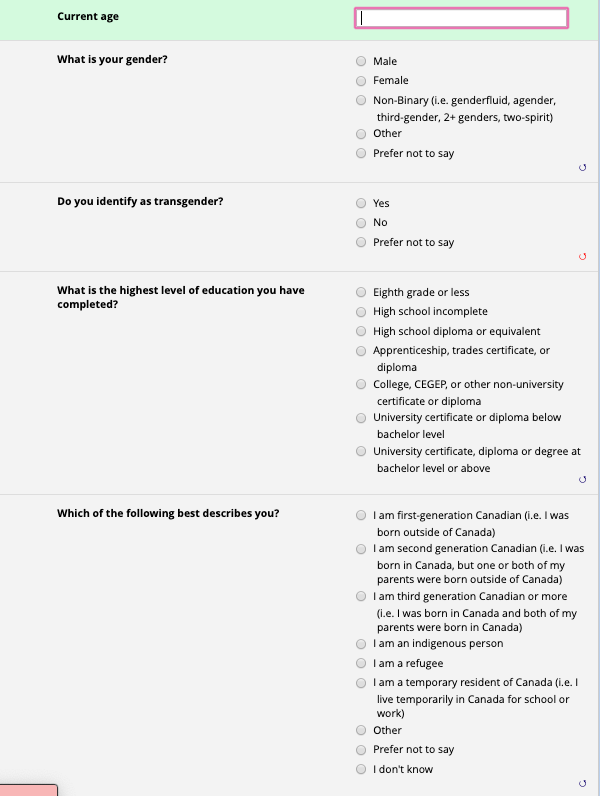


Past Experiences with Mental Health Referrals


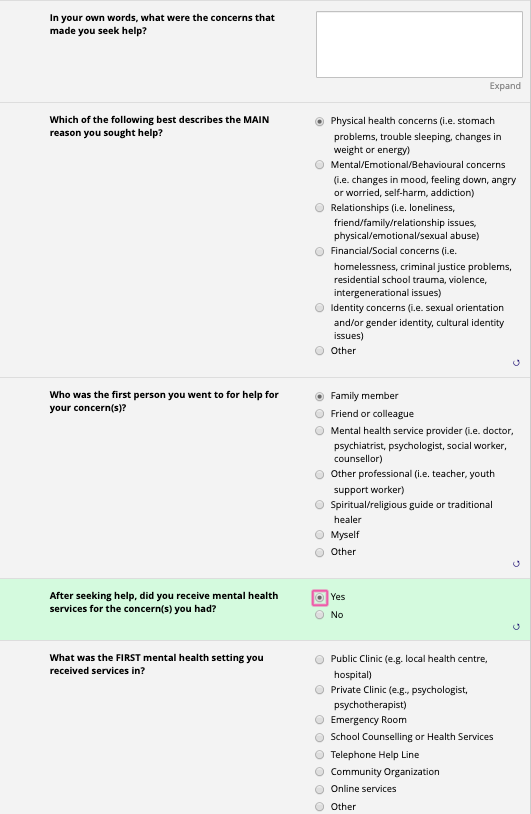


Views on an Online Self-Referral Tool


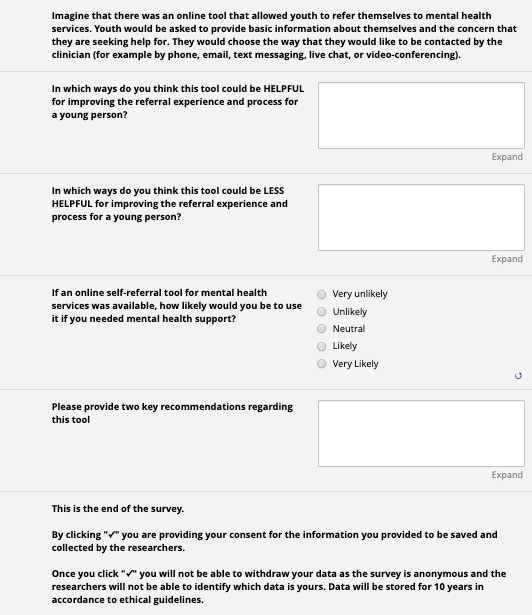


End Screen


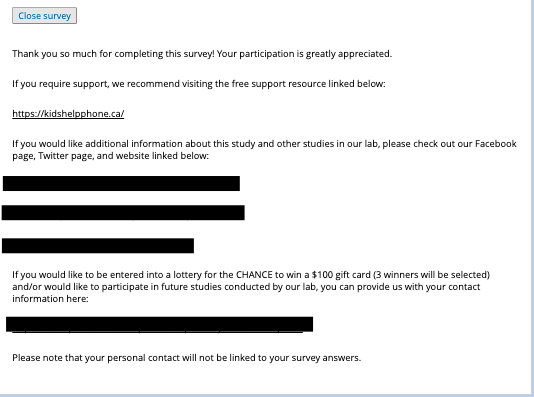


Follow-Up

#
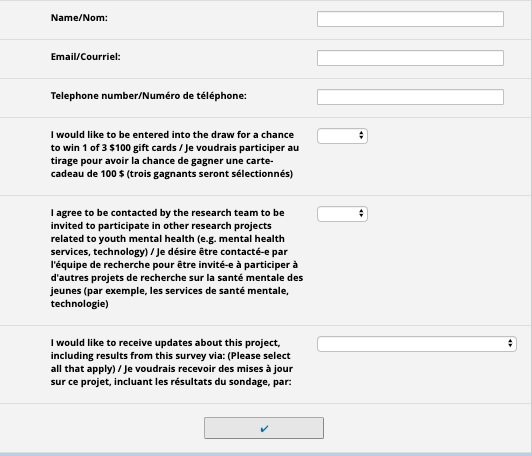

Supplement: Multimedia Appendix 1 [file resprot_v9i3e16945_app1.docx]
