# Supplementary material for: Youth Experiences With Referrals to Mental Health Services in Canada: Protocol for a Web-Based Cross-Sectional Survey Study
Source: JMIR Res Protoc. 2020 Mar 24;9(3):e16945. doi: 10.2196/16945 (PMC7139421; doi:10.2196/16945)
Supplement: Multimedia Appendix 2 [file resprot_v9i3e16945_app2.docx]

Appendix B: Questionnaire

**SURVEY WRITTEN COMPONENTS: PRE-SCREENING**

| Survey Introduction | |
| --- | --- |
| English | French |
| Thank you for your interest in our survey!  We are the Youth Mental Health and Technology Lab located at the University of Montreal Hospital Research Centre.  We are conducting a survey about how youth access mental health services in Canada. This survey is part of a research project called "Youth Experiences with Mental Health Referrals in Canada: An Online Survey" led by Dr. Shalini Lal.  Specifically, the survey is about the experiences that youth have with the referral process to mental health services. A referral is when someone directs you to a service that might be appropriate for your needs. Referrals can be given from family doctors, counsellors, or in some cases you can even refer yourself to a service.  We would appreciate learning more about your personal experiences with referrals to mental health services, including the pathways you took to access services, and the barriers you experienced when trying to access services. We also want to know what you think about the idea of using technology to facilitate the self-referral process. The information that you provide will contribute to knowledge on how to improve the mental health referral process for young people in Canada.  The survey takes about 10-15 minutes to complete. Your participation in this survey is completely voluntary. Any answers you give will remain confidential as the survey is anonymous and we will not be able to identify you.  At the end of the survey you will have the option to be entered into a lottery for a CHANCE to win a $100 gift card (3 winners will be selected!).  If you have any questions please feel free to contact:  Danielle Starcevic, Research Assistant  E-mail: danielle.starcevic.crchum@gmail.com  We will begin with a few short questions to determine if you are eligible to participate. | Merci de votre intérêt pour notre sondage!  Nous sommes le Laboratoire Santé mentale des jeunes et technologies situé au Centre de recherche du Centre hospitalier de l'Université de Montréal.  Nous réalisons un sondage sur les modalités d'accès aux services de santé mentale chez les jeunes au Canada. Le sondage fait partie d'un projet de recherche intitulé "Sondage en ligne sur les expériences des jeunes quant à l'aiguillage vers des services de santé mentale au Canada" et est dirigé par Dre Shalini Lal.  Plus précisément, le sondage porte sur les expériences des jeunes par rapport au processus de référence vers les services de santé mentale. On parle de "référence" ou "d'aiguillage" lorsque quelqu'un vous dirige vers un service qui pourrait correspondre à vos besoins. Les références peuvent être complétées par des médecins de famille, des intervenants ou, dans certains cas, vous pouvez vous référer vous-même vers un service.  Nous aimerions en apprendre davantage sur vos expériences personnelles d'aiguillage vers des services de santé mentale, y compris les parcours que vous avez suivis pour accéder aux services et les obstacles que vous avez rencontrés en chemin. Nous aimerions également connaître votre opinion sur l'idée d'utiliser la technologie pour faciliter le processus d'autoréférence. L'information que vous fournirez contribuera à améliorer le processus de référence vers les services de santé mentale au Canada.  Le sondage prend environ 10 à 15 minutes à remplir. Votre participation est entièrement volontaire. Vos réponses demeureront confidentielles puisque le sondage est anonyme et que nous ne pourrons donc pas vous identifier.  À la fin du sondage vous pourrez participer à un tirage pour la CHANCE de gagner une carte-cadeau de 100$ (3 gagnants seront sélectionnés!).  Si vous avez des questions, n'hésitez pas à contacter:  Danielle Starcevic, Assistante de recherche  courriel: danielle.starcevic.crchum@gmail.com  Nous allons commencer avec quelques questions courtes pour déterminer si vous êtes éligible à participer au sondage. |

| Survey Consent Form | |
| --- | --- |
| English | French |
| Thank you, you are eligible to participate! Before we begin the survey please read the following consent from.  UNIVERSITY OF MONTREAL HOSPITAL RESEARCH CENTRE  Consent to Participate in Research  The following form briefly explains the study in which you are being asked to participate in. Please read this form carefully and contact the investigator(s) listed below if you have any questions before you provide your consent to participate.  Title of the Research Project:  Youth Experiences with Mental Health Referrals in Canada: An Online Survey  Principal Investigator:  Shalini Lal, PhD  Co-Investigator:  Rebecca Fuhrer, PhD  Research Assistant:  Danielle Starcevic, BSc  Voluntary participation:  Your participation in this survey is completely voluntary. You have the right to withdraw your participation at any time before clicking submit (indicated by the “✓”) at the end of the survey by simply exiting any page. None of your answers will be collected unless you click "✓". This survey is anonymous; this means that once you submit your answers the researchers will not be able to identify which data is yours and your data cannot be withdrawn.  By clicking "✓" at the end of this survey you are providing your consent to participate. You are not waiving any of your legal rights as a research participant by consenting to participate.  What you are being asked to do:  You are being asked to complete this online survey as accurately as possible. This survey takes approximately 10-15 minutes to complete. The survey includes questions about socio-demographic information (age, gender, ethnicity, education status, etc.), past experiences accessing mental health services/referrals in Canada, and your opinions on an online-self referral service.  You may also choose to provide us with your name and contact information at the end of this survey if you are interested in participating in future studies and/or being entered into the gift card lottery. This information will be collected on a separate page from the survey and will not be linked to the information you provide in the survey.  Benefits:  Participants will have the CHANCE to win a $100 gift card (three winners will be selected). The data collected in this survey will contribute to the improvement of mental health care services for youth in Canada.  Risks:  This survey is anonymous and the information that you will provide will be non-identifiable. Your Internet Protocol (IP) address will be collected by the survey tool but the researchers will not have access to this information. There is a small risk of interference from third-parties and the information you provide in the survey could be traced back to you through your IP address. This survey is hosted on a secure server at the University of Montreal Hospital Research Centre in order to minimize this risk.  The questions in this survey are mainly about accessing mental health services. Some of these questions may make you uncomfortable, upset, or you may not want to answer them. You may skip any question(s) you do not want to answer or stop your participation at any time by simply exiting the survey.  If you require support during or after the survey is complete we recommend visiting the free support resource listed below:  https://kidshelpphone.ca/  How your information will be protected and stored:  Your privacy and confidentiality will be protected throughout this study. All electronic data (results of the survey and contact information) will be stored on a password protected computer in a secure office at the University of Montreal Hospital Research Centre. Only members of the research team will have access to this data. Any future publications of the data will summarize information and individual responses will not be shared with anyone outside of the research team. When the research project is done, the data will be stored for up to 10 years before being destroyed.  Questions:  If you have any questions about this research, please contact:  Danielle Starcevic, Research Assistant  E-Mail: danielle.starcevic.crchum@gmail.com  This survey study has been approved by the University of Montreal Hospital Centre Research Ethics Board (Project #18.255). If you have questions about your rights or treatment as a research participant or if you have any complaints regarding this study, you can contact the service quality and complaints commissioner of the University of Montreal Hospital Centre at: Contact: 514-890-8484. | Merci! Vous êtes éligible à participer au sondage! Avant de commencer, svp lire le formulaire de consentement ci-après:  CENTRE DE RECHERCHE DU CENTRE HOSPITALIER DE L'UNIVERSITÉ DE MONTRÉAL  Consentement à participer à une recherche  Le présent formulaire explique brièvement l'étude pour laquelle votre participation est sollicitée. Avant d'accepter de participer à la recherche, veuillez lire attentivement ce formulaire et contacter le(s) chercheur(s) identifié(s) ci-dessous si vous avez des questions.  Titre du projet de recherche:  Sondage en ligne sur les expériences des jeunes quant à l'aiguillage vers des services de santé mentale au Canada  Chercheuse principale:  Shalini Lal, Ph.D.  Co-chercheuse:  Rebecca Fuhrer, PhD  Assistant de recherche:  Danielle Starcevic, B.Sc.  Participation volontaire:  Votre participation à ce sondage est entièrement volontaire. Vous avez le droit de vous retirer à n'importe quel moment avant d'avoir cliqué sur soumettre (indiqué par "✓") à la fin du sondage, simplement en quittant la page de votre fureteur. Aucune de vos réponses ne sera conservée à moins que vous appuyiez sur "✓". Ce sondage est anonyme; après avoir envoyé vos réponses, les chercheurs ne pourront plus identifier lesquelles vous appartiennent et vous ne pourrez plus retirer vos informations.  En cliquant sur "✓" à la fin du sondage, vous consentez à y participer. Vous ne renoncez pas à vos droits en tant que participant de recherche en consentant à y participer.  Ce qui vous est demandé:  Nous vous demandons de remplir ce sondage en ligne de la manière la plus exacte possible. Le sondage prend de 10 à 15 minutes à remplir. Le sondage comprend des questions sur des informations d'ordre sociodémographique (âge, sexe, origines ethniques, niveau d'éducation, etc.), sur vos expériences quant à l'accès aux services de santé mentale au Canada, ainsi que visant à connaître votre opinion sur un service d'autoréférence en ligne.  Vous avez également le choix de nous fournir votre nom et vos coordonnées à la fin du sondage si vous êtes intéressé-e à participer à d'autres études ou à un tirage pour des cartes-cadeaux. Vos coordonnées seront recueillies sur une autre page que celle du sondage et ne seront pas mises en lien avec l'information que vous aurez fournie dans le sondage.  Bienfaits:  Les participants auront la chance de gagner une carte-cadeau de 100$ (trois gagnants seront sélectionnés). Les données du sondage contribueront à l'amélioration des services de santé mentale pour les jeunes au Canada.  Risques:  Le sondage est anonyme et l'information que vous fournissez est non identifiable. Votre numéro internet (adresse IP) sera collecté par la plateforme de sondage mais les chercheurs n'auront pas accès à cette information. Il existe un faible risque d'interférence de la part de tiers et l'information que vous avez fournie pourrait être retracée grâce à votre adresse IP. Le sondage est hébergé sur un serveur sécurisé au Centre de recherche du Centre hospitalier de l'Université de Montréal afin de minimiser ce risque.  Les questions de ce sondage portent principalement sur l'accès aux services de santé mentale. Certaines questions pourraient susciter un malaise ou un inconfort ou vous pourrez souhaiter ne pas y répondre. Vous pouvez sauter n'importe quelle question si vous ne souhaitez pas y répondre et mettre fin à votre participation à n'importe quel moment simplement en quittant le sondage.  Si vous avez besoin de soutien durant le sondage ou après le sondage nous vous encourageons à contacter la ressource d'aide gratuite ci-dessous:  https://jeunessejecoute.ca/  Mesures de protection et de conservation de vos données:  Votre vie privée et votre droit à la confidentialité seront protégés tout au long de cette étude. Toutes les données électroniques (résultats du sondage et coordonnées) seront sauvegardées dans un ordinateur protégé par un mot de passe dans un bureau sécurisé au Centre de recherche du Centre hospitalier de l'Université de Montréal. Seuls les membres de l'équipe de recherche auront accès à ces données. Toutes publications futures portant sur les données synthétiseront ces informations et les réponses individuelles ne seront partagées avec quiconque en dehors de l'équipe de recherche. À la fin du projet, les données seront conservées pour une durée maximale de 10 ans avant d'être détruites.  Question:  Si vous avez des questions concernant cette recherche, veuillez contacter:  Danielle Starcevic, Assistant de recherche  Courriel: danielle.starcevic.crchum@gmail.com  Cette étude a été approuvée par le comité d'éthique de la recherche du Centre hospitalier de l'Université de Montréal (Projet #18.225). Si vous avez des questions concernant vos droits ou votre traitement en tant que participant-e, ou si vous souhaitez déposer une plainte par rapport à cette étude, vous pouvez contacter le commissaire local aux plaintes et à la qualité des services du Centre hospitalier de l'Université de Montréal: 514-890-8484. |

| Survey Ineligibility | |
| --- | --- |
| English | French |
| Thank you so much for your interest in our survey. You are not eligible for this study at this time.  Please feel free to contact the following researcher if you have any questions:  Danielle Starcevic, Research Assistant  E-mail: danielle.starcevic.crchum@gmail.com  Thank you for your time.  You can leave this survey by simply exiting the page. | Merci beaucoup de votre intérêt pour notre sondage. Vous n'êtes pas éligible à y participer à l'heure actuelle.  N'hésitez pas à contacter la chercheuse ci-dessous si vous avez des questions:  Danielle Starcevic, Assistant de recherche  courriel: danielle.starcevic.crchum@gmail.com  Merci pour le temps que vous y avez consacré.  Vous pouvez quitter le sondage simplement en quittant la page. |

**SURVEY WRITTEN COMPONENTS: POST-SURVEY**

| Survey End Screen | |
| --- | --- |
| English | French |
| Thank you so much for completing this survey! Your participation is greatly appreciated.  If you require support, we recommend visiting the free support resource linked below:  <https://kidshelpphone.ca/>  If you would like additional information about this study and other studies in our lab, please check out our Facebook page, Twitter page, and website linked below:  Facebook: <https://www.facebook.com/ymhtech/>  Twitter: https://twitter.com/ymhtech_smjtech  Website: http://www.ymhtech.com/  If you would like to be entered into a lottery for the CHANCE to win a $100 gift card (3 winners will be selected) and/or would like to participate in future studies conducted by our lab, you can provide us with your contact information here:  <https://redcap.chumontreal.qc.ca/redcap/surveys/?s=3AHNWJXTH3>  Please note that your personal contact will not be linked to your survey answers. | Merci beaucoup d’avoir complété ce sondage! Votre participation est grandement appréciée.  Si vous avez besoin de soutien, nous vous encourageons à contacter la ressource d’aide gratuite ci-dessous:  <https://jeunessejecoute.ca/>  Si vous aimeriez recevoir plus d’informations sur cette étude et d’autres études entreprises par notre Labo, veuillez consulter notre page Facebook, notre page Twitter et notre site web:  Facebook: <https://www.facebook.com/ymhtech/>  Twitter: <https://twitter.com/ymhtech_smjtech>  Site web: http://[www.smjtechno.com](http://www.smjtechno.com/)  Si vous aimeriez participer au tirage pour la CHANCE de gagner une carte-cadeau de 100$ (3 gagnants seront sélectionnés) et/ou vous aimeriez participer à d’autres études entreprises par notre Labo veuillez partager vos coordonnées avec nous:  <https://redcap.chumontreal.qc.ca/redcap/surveys/?s=3AHNWJXTH3>  Veuillez noter que vos coordonnées personnelles ne seront pas liées à vos réponses au sondage. |

| Optional Survey Contact Information Form* | |
| --- | --- |
| English | French |
| Name: | Nom: |
| Email: | Courriel: |
| Telephone number: | Numéro de téléphone: |
| I would like to be entered into the draw for a chance to win 1 of 3 $100 gift cards  1, Yes  2, No | Je voudrais participer au tirage pour avoir la chance de gagner une carte-cadeau de 100 $ (trois gagnants seront sélectionnés)  1, Oui  2, Non |
| I agree to be contacted by the research team to be invited to participate in other research projects related to youth mental health (e.g. mental health services, technology)  1, Yes  2, No | Je désire être contacté-e par l’équipe de recherche pour être invité-e à participer à d’autres projets de recherche sur la santé mentale des jeunes (par exemple, les services de santé mentale, technologie)  1, Oui  2, Non |
| I would like to receive updates about this project, including results from this survey via: (Please select all that apply)  1, Email  2, Text (SMS)  3, I would not like to receive updates | Je voudrais recevoir des mises à jour sur ce projet, incluant les résultats du sondage, par:  1, Courriel  2, Texte (SMS)  3, Je ne voudrais pas recevoir de mises à jour |

*Note: This instrument was collected as a separate project in REDCap and is not linked with the data from the primary survey

| Facebook Recruitment Post (to be accompanied by previously approved recruitment flyers) | |
| --- | --- |
| English | French |
| Hello!    The Youth Mental Health and Technology lab at the University of Montreal Hospital Research Centre is seeking volunteers between the ages of 17-30 to participate in an anonymous survey about youth’s experiences with referrals to mental health services in Canada. It will take 10-15 mins to complete, and is about young people’s experiences seeking help for mental health concerns in Ontario, Quebec, and British Columbia. All participants will have the opportunity to enter a draw to win ONE of THREE $100 gift cards. Please see below for more details and to access the link to the survey!  English Survey: https://redcap.chumontreal.qc.ca/redcap/surveys/?s=AXTK9E3K7D  French Survey: https://redcap.chumontreal.qc.ca/redcap/surveys/?s=XWWX7RMHPF | Bonjour!    Le laboratoire de santé mentale des jeunes et technologies de Centre de recherche du Centre hospitalier de l’Université de Montréal (CRCHUM) est à la recherche de bénévoles âgés de 17 à 30 ans pour participer à un sondage anonyme sur les expériences des jeunes par rapport à l’aiguillage vers les services de santé mentale au Canada. Le sondage dure entre 10 à 15 minutes et porte sur les expériences des jeunes du Québec, de l’Ontario et de la Colombie-Britannique cherchant de l’aide pour des préoccupations liées à leur santé mentale. Tous les participants auront l’opportunité de gagner une carte-cadeau de 100 $ (trois gagnants seront sélectionnés). Pour plus des détails et pour accéder au sondage, svp voir ci-dessous.  Sondage en Français: https://redcap.chumontreal.qc.ca/redcap/surveys/?s=XWWX7RMHPF  Sondage en Anglais: https://redcap.chumontreal.qc.ca/redcap/surveys/?s=AXTK9E3K7D |

**SURVEY QUESTIONS**

| Question # | English Survey Revised | French Survey Revised |
| --- | --- | --- |
| SCREENING QUESTIONS | | |
| 1 | How old are you?  Below 17  17  18  19  20  21  22  23  24  25  26  27  28  29  30  Above 30 | Quel âge avez-vous?  Moins de 17 ans  17  18  19  20  21  22  23  24  25  26  27  28  29  30  Plus de 30 ans |
| 2 | Have you used or tried to use  mental health services in the past 5 years?  Yes  No | Au cours des 5 dernières années, avez-vous reçu ou avez-vous tenté de recevoir des services de santé mentale?  Oui  Non |
| 3 | Did you use or try to use mental health services in Quebec, Ontario, or British Columbia?  Yes  No | Avez-vous reçu ou avez-vous tenté de recevoir des services de santé mentale au Québec, en Ontario ou en Colombie-Britannique?  Oui  Non |
| 4 | I have read the form above and I give my consent to participate  Yes  No | J'ai lu le formulaire ci-dessus et je consens à participer à ce sondage  Oui  Non |
| DEMOGRAPHIC QUESTIONS | | |
| 5 | Current age | Âge actuel |
| 6 | What is your gender?  1, Male  2, Female  3, Non-Binary (i.e. genderfluid, agender,  third-gender, 2+ genders, two-spirit)  4, Other  5, Prefer not to say | Vous êtes de sexe:  1, Masculin  2, Féminin  3, Non binaire (ex: entre-deux, agenre, troisième genre, 2+, bi-spirituel)  4, Autre  5, Je préfère ne pas répondre |
| 7 | Do you identify as transgender?  1, Yes  2, No  3, Prefer not to say | Vous identifiez-vous en tant que transgenre?  1, Oui  2, Non  3, Je préfère ne pas répondre |
| 8 | What is the highest level of education you have completed?  1, Eighth grade or less  2, High school incomplete  3, High school diploma or equivalent  4, Apprenticeship, trades certificate, or diploma  5, College, CEGEP, or other non-university certificate or diploma  6, University certificate or diploma below bachelor level  7, University certificate, diploma or degree at bachelor level or above | Quel est le plus haut niveau d’éducation que vous avez atteint?  1, 8ème année ou moins  2, Études secondaires incomplètes  3, Diplôme d’études secondaires ou équivalent  4, Certificat ou diplôme d'apprenti ou d'une école de métiers  5, Certificat ou diplôme d'un collège, d'un cégep ou d'un autre établissement non universitaire  6, Certificat ou diplôme universitaire inférieur au baccalauréat  7, Certificat, diplôme ou grade universitaire au niveau du baccalauréat ou supérieur |
| 9 | Which of the following best describes you?  1, I am first-generation Canadian  (i.e. I was born outside of Canada)  2, I am second generation Canadian  (i.e. I was born in Canada,  but one or both of my parents were born outside of Canada)  3, I am third generation Canadian or more  (i.e. I was born in Canada and  both of my parents were born in Canada)  4, I am an indigenous person  5, I am a refugee  6, I am a temporary resident of Canada  (i.e. I live temporarily in Canada for school or work)  7, Other (Please specify)  8, Prefer not to say  9, I don't know | Lequel des énoncés suivants vous décrit le mieux?  1, Je suis Canadien-ne de première génération (c-à-d, je suis né-e à l'extérieur du Canada)  2, Je suis Canadien-ne de deuxième génération (c-à-d, je suis né-e au Canada mais un de mes parents, ou mes deux parents, sont nés à l'extérieur du Canada)  3, Je suis Canadien-ne de troisième génération ou plus (c-à-d, je suis né-e au Canada et mes deux parents sont nés au Canada)  4, Je suis une personne autochtone  5, J'ai le statut de réfugié  6, Je suis résident temporaire au Canada (c-à-d, j'habite au Canada de manière temporaire pour les études ou le travail)  7, Autre (Veuillez spécifier)  8, Je préfère ne pas répondre  9, Je ne sais pas |
| 10 | What are the ethnic or cultural origins of your ancestors?  [Please check all that apply] ^[[1]](#footnote-1)^  (Note: Ethnic origins refer to an individual's roots and not to an individual's citizenship,  nationality, language, or place of birth.  Ancestors are relatives more distant than a grandparent)  1, Oceania origins  (e.g. Australian, New Zealander, Fijian, Maori, Polynesian, Samoan, Hawaiian, and other Oceanic/Pacific origins)  2, North American Indigenous origins  (e.g. First Nations, Metis, Inuit, and other North American Indigenous origins)  3, European origins  (e.g. English, Scottish, French, Irish, German, Italian, Ukranian, Dutch, Polish, Russian and other British Isles, French, Northern, Western, Eastern, and Southern European origins)  4, Latin, Central, or South American origins  (e.g. Mexican, Colombian, Guyanese, Salvadorean, Chilean, Peruvian, Brazilian, Argentinian, Venezuelan, Ecuadorian, and other Latin, Central and South American origins)  5, Caribbean origins  (e.g. Jamaican, Haitian, Trinidadian/Toboganian, Barbadian, Cuban, Dominican, Puerto Rican, Guadeloupean, Martinican, Bahamian, and other Caribbean origins)  6, Other North American origins  (e.g. Canadian, American, Québécois, Acadian, Ontarian, Nova Scotian, New Brunswicker, Newfoundlander, and Other North American origins)  7, African origins  (e.g. Moroccan, Egyptian, Algerian, Somali, Nigerian, Ethiopian, South African, Congolese, Berber, Ghanian, and other Central, West, North, South, and East African origins)  8, Asian origins  (e.g. Chinese, East Indian, Filipino, Vietnamese, Lebanese, Pakistani, Iranian, Korean, Sri Lankan, Japanese, and other West Central, Middle Eastern, South, East, and Southeast Asian origins)  10, Other (Please specify)  9, Prefer not to say  11, Prefer to self-identify  12, I don't know | Quelles sont les origines ethniques ou culturelles de vos ancêtres? [Vous pouvez sélectionner plus d'une réponse]  (Précisions: Les origines ethniques se rapportent aux  “racines” d’une personne, d’un individu et non à sa citoyenneté, sa nationalité, sa langue ou son lieu de naissance. Les ancêtres sont des parents éloignés des grands-parents)  1, Océanie  (ex: Australien-ne, Néo-Zélandais, Fidjien-ne, Maori, Polynésien-ne, Samoan, Hawaïen-ne, et autres origines Océanique/îles du Pacifique)  2, Autochtones d'Amérique du Nord  (ex: Premières Nations, Métis-se, Inuit et autres origines autochtones nord-américains)  3, Europe  (ex: Anglais-e, Écossais-e, Français-e, Italien-ne, Ukrainien-ne, Néerlandais-e, Polonais-e, Russe, et autres origines Îles britanniques, Français, Europe de l'Est, Europe de l'Ouest, Sud de l'Europe, Nord de l'Europe)  4, Amérique latine, Amérique centrale ou Amérique du Sud  (ex: Mexicain-e, Colombien-ne, Guyanais-e, Salvadorien-ne, Chilien-ne, Péruvien-ne, Brésilien-ne, Argentin-e, Vénézuélien-ne, Équatorien-ne, et autres origines Latin, Central, et Sud-Americain)  5, Caraïbes  (ex: Jamaïcain-e, Haïtien-ne, Trinidadien-ne/Tobogonien-ne, Barbadien-ne, Cubain-e, Dominicain-e, Portoricain-e, Guadeloupéen-ne, Martiniquais-e, Bahamien-e, et autres origines Caraïbes)  6, Autres origines nord-américaines  (ex: Canadien-ne, Américain-e, Québécois-e, Acadien-ne, Ontarien-ne, Néo-Écossais-e, Nouveau-Brunswick, Terre-Neuve, et autres origines Nord-Américain)  7, Afrique  (ex: Marocain-e, Égyptien-ne, Algérien-ne, Somalien-ne, Nigérien-ne, Éthiopien-ne, Sud-Africain-e, Congolais-e, Berbère, Ghanéen-ne, et autres origines Nord, Est, Ouest, Centrale et Sud-Africain)  8, Asie  (ex: Chinois-e, Est-Indien-ne, Philippin-e, Vietnamien-ne, Libanais-e, Pakistanais-e, Iranien-ne, Coréen-ne, Sri Lankais-e, Japonaise-e, et autres origines Asie du Ouest-Centrale, Moyen Orient, Asie du Sud, Asie d’Est, et Asie du Sud-Est)  10, Autres (Veuillez spécifier)  9, Je préfère ne pas répondre  11, Je préfère m'auto-identifier  12, Je ne sais pas |
| 11 | In your own words, what are the ethnic  or cultural origins of your ancestors? | Quelles sont les origines ethniques ou culturelles de vos ancêtres (dans vos propres mots)? |
| EXPERIENCES WITH MENTAL HEALTH SERVICES | | |
|  | Please answer the following questions about the  FIRST TIME you tried seeking help for  a mental health concern in the past 5 years. | Veuillez répondre aux questions suivantes par rapport à la PREMIÈRE FOIS que vous avez cherché de l'aide pour une préoccupation liée à la santé mentale, durant les 5 dernières années |
| 12 | How old were you at the time? | Quel âge aviez-vous à ce moment-là? |
| 13 | What province did you live in at the time?  0, Ontario  1, Quebec  2, British Columbia | Dans quelle province habitiez-vous à ce moment-là?  0, Ontario  1, Québec  2, Colombie-Britannique |
| 14 | How long had you lived in Canada for at the time?  1, Less than a year  2, 1-5 years  3, 6-10 years  4, 11+ years | Depuis combien de temps habitiez-vous au Canada à ce moment-là?  1, Moins d'un an  2, 1 à 5 ans  3, 6 à 10 ans  4, Plus de 11 ans |
| 15 | Which of the following best describes  where you lived at the time?  1, Large population centre  (population of 100,000 or more)  2, Medium population centre  (population between 30,000 to 99, 999)  3, Small population centre  (population between 1,000 to 29,999)  4, Rural  (Population of less than 1,000) | Laquelle des réponses ci-dessous décrit le mieux votre lieu de résidence à ce moment-là?  1, Grand centre de population urbain  (plus de 100 000 habitants)  2, Moyen centre de population  (entre 30 000 et 99 999 habitants)  3, Petit centre de population  (entre 1 000 et 29 999 habitants)  4, Région rurale  (moins de 1 000 habitants) |
| 16 | Which of the following best describes your  living situation at the time?  1, I was living with my family or my guardian  2, I was living alone  3, I was living with one or more roommates  4, I was living with my spouse  5, I was living in supported housing  6, I did not have a fixed address  (e.g. I was living temporarily with relatives or friends, in shelters/on the street, I was couch-surfing)  7, Other (Please specify) | Laquelle des réponses ci-dessous décrit le mieux votre situation en logement à ce moment-là?  1, J'habitais avec ma famille ou mon tuteur/ma tutrice  2, J'habitais seul-e  3, J'habitais avec un/des colocataire-s  4, J'habitais avec mon époux/épouse  5, J'habitais dans un logement supervisé  6, Je n'avais pas d'adresse fixe  (ex: J'habitais temporairement chez de la parenté ou des amis, j'habitais dans les refuges/dans la rue, je faisais du squat de canapé/couch-surfing)  7, Autre (Veuillez spécifier) |
| 17 | Which of the following best describes  your main activity at the time?  1, Student  2, Employed full-time  3, Employed part-time  4, Not employed/not in school  5, Volunteer  6, Other (Please specify) | Laquelle des réponses ci-dessous décrit le mieux votre principale activité à ce moment-là?  1, Étudiant-e  2, Employé-e à temps plein  3, Employé-e à temps partiel  4, Pas d'emploi/pas inscrit-e aux études  5, Bénévole  6, Autre (Veuillez spécifier) |
| 18 | Which of the following best describes your approximate annual household income at the time?  1, Less than $47,000 per year  2, $47,000 - $93, 000 per year  3, $94,000 - $145,000 per year  4, $146,000 - $206,000 per year  5, Over $206,000 per year  6, I don’t know  7, Prefer not to say  8, Other (Please specify) | Lequel des énoncés suivants décrit le mieux votre revenu annuel familial à ce moment-là?  1, Moins de 47 000$ par année  2, 47 000$ - 93 000$ par année  3, 94 000$ - 145 000$ par année  4, 146 000$ - 206 000$ par année  5, Plus de 206 000$ par année  6, Je ne sais pas  7, Je préfère ne pas répondre,  8, Autre (Veuillez spécifier) |
| 19 | In your own words, what were  the concerns that made you seek help? | Dans vos propres mots, décrivez les préoccupations qui vont ont motivé-e à chercher de l’aide. |
| 20 | Which of the following best describes the MAIN reason you sought help?  1, Physical health concerns  (i.e. stomach problems, trouble sleeping, changes in weight or energy)  2, Mental/Emotional/Behavioural concerns (i.e. changes in mood, feeling down, angry or worried, self-harm, addiction)  3, Relationships  (i.e. loneliness, friend/family/relationship issues, physical/emotional/sexual abuse)  4, Financial/Social concerns  (i.e. homelessness, criminal justice problems, residential school trauma, violence, intergenerational issues)  5, Identity concerns  (i.e. sexual orientation and/or gender identity, cultural identity issues)  6, Other (Please specify) | Laquelle des réponses ci-dessous décrit le mieux la raison PRINCIPALE pour laquelle vous avez cherché de l'aide?  1, Santé physique (ex: problèmes d'estomac, sommeil difficile, changements liés au poids/énergie)  2, Santé psychologique, émotions et comportement (ex: changements liés à l'humeur, sentiments de déprime, colère, inquiétude, automutilation, dépendances)  3, Relations personnelles  (ex: solitude, problèmes liés aux amis, famille ou partenaire, abus physiques, psychologiques ou sexuels)  4, Économique/Social  (ex: itinérance, problèmes avec la justice, traumatismes liés aux pensionnats indiens, violence, problèmes intergénérationnels)  5, Identité  (ex: questions liées à l'orientation sexuelle, identité de genre, identité culturelle)  6, Autre (Veuillez spécifier) |
| 21** | Who was the first person you went to  for help for your concern(s)?  1, Family member  2, Friend or colleague  3, Mental health service provider  (i.e. doctor, psychiatrist, psychologist,  social worker, counsellor)  4, Other professional  (i.e. teacher, youth support worker)  5, Spiritual/religious guide or traditional healer  6, Myself  7, Other (Please specify) | Qui avez-vous approché, en premier lieu, pour vous aider à répondre à vos préoccupations?  1, Un membre de votre famille  2, Un ami ou un collègue  3, Un-e professionnel-le de la santé mentale  (ex: médecin, psychiatre, psychologue, travailleur/se social-e, thérapeute)  4, Un-e autre professionnel-le  (ex: enseignant-e, intervenant-e jeunesse)  5, Un-e chef-fe spirituel-le ou un-e guérisseur/se traditionnel-le  6, Moi-Même  7, Autre (Veuillez spécifier) |
| 22 | After seeking help, did you receive mental health services for the concern(s) you had?  Yes  No | Après avoir cherché de l'aide, est-ce que vous avez reçu des services de santé mentale en lien avec vos préoccupations?  Oui  Non |
| 23** If answer to Q19 is NO | Which of the following best describes the MAIN obstacle that kept you from receiving mental health services for the concern(s) you had?  1, Personal stigma  (i.e. worries/anxiety about judgement from others,  being a burden, the help-seeking process)  2, Social stigma and discrimination  (i.e. racism, sexism, homophobia, transphobia,  classism, judgement/insensitivity from others)  3, Confidentiality issues  (i.e. concerns about others finding out/  service provider telling others)  4, Lack of knowledge  (i.e. about mental health and mental illness,  about mental health services available)  5, Concerns about the service provider  (i.e. worried they wouldn't help/meet my needs,  uncomfortable talking to them)  6, Wait times  (i.e. long wait lists, service provider was busy)  7, Accessibility issues  (i.e. service location, cost,  difficulty getting in contact with service providers)  8, Communication issues  (i.e. language barriers)  9, Preferred help from other sources  (i.e. myself, friends, family, religious leaders)  10, Disability (e.g, related to physical mobility, visual impairment, auditory and speech impairment)  11, Scheduling conflicts  12, Other (Please specify)  13, I'm not sure  14, I did not experience any obstacles | Laquelle des options ci-dessous décrit le mieux le PRINCIPAL obstacle qui vous a empêché-e de recevoir des services de santé mentale en lien avec vos préoccupations?  1, Stigmatisation personnelle  (ex: inquiétude/anxiété liée à la possibilité d'être jugé-e ou de représenter un fardeau pour les autres ou par rapport au processus de demande d'aide)  2, Stigmatisation et discrimination sociales  (ex: racisme, sexisme, homophobie, transphobie, préjugés de classe, jugement/manque de sensibilité de la part d'autrui)  3, Inquiétudes liées à la confidentialité  (ex: soucis que d'autres personnes l'apprennent ou que le/la professionnel-le en parle à d'autres)  4, Connaissances insuffisantes  (ex: à propos de la santé mentale et de la maladie mentale, à propos des services disponibles en santé mentale)  5, Inquiétudes liées au/à la professionnel-le  (ex: inquiétude qu'il/elle ne pourrait pas m'aider/répondre à mes besoins, inconfort dans le fait de parler avec lui/elle)  6, Temps d'attente  (ex: longues listes d'attente, manque de disponibilité du/de la professionnel-le)  7, Accessibilité  (ex: emplacement des services, coût, difficulté à rejoindre le/la professionnel-le)  8, Communication  (ex: barrières linguistiques)  9, Préféré trouver de l'aide ailleurs  (ex: de moi-même, de mes amis, de ma famille, d'un-e chef-fe religieux/se)  10, Incapacité (ex: liée à la mobilité physique, déficience visuelle ou auditive, troubles de parole et de langage)  11, Conflits d'horaire  12, Autre (Veuillez spécifier)  13, Je ne sais pas  14, Je n'ai pas rencontré d'obstacles |
| 24*** If answer to Q19 is YES | What was the FIRST mental health setting you received services in?  1, Public Clinic (e.g. local health centre, hospital)  2, Private Clinic (e.g., psychologist, psychotherapist)  3, Emergency Room  4, School Counselling or Health Services  5, Telephone Help Line  6, Community Organization  7, Online services  8, Other (Please specify) | À quel service vous êtes-vous présenté-e EN PREMIER?  1, Clinique Publique  2, Clinique Privée (ex: psychologue, psychothérapeute)  3, Urgence  4, Services de santé de l'école  5, Service d'aide téléphonique  6, Organisme communautaire  7, Services d'aide en ligne  8, Autre (Veuillez spécifier) |
| 25 | Who was the FIRST mental health service provider that you received mental health services from?  1, Doctor  2, Counsellor  3, Psychologist  4, Psychiatrist  5, Other mental health service provider  (e.g. nurse, social worker...)  6, Peer  7, I'm not sure  8, Other (Please specify) | De quel type de professionnel-le de santé mentale avez-vous reçu des services EN PREMIER?  1, Médecin  2, Thérapeute  3, Psychologue  4, Psychiatre  5, Autre professionnel-le de la santé mentale (ex: infirmière, travailleur/se social-e...)  6, Pair-aidant  7, Je ne sais pas  8, Autre (Veuillez spécifier) |
| 26 | Which type of service(s) did you receive from this mental health service provider? [Please check all that apply]  1, Information  2, Medication  3, Counselling/Therapy  4, Peer support  5, Other (Please specify) | Quel(s) type(s) de service(s) avez-vous reçu(s) de la part de ce fournisseur de services en santé mentale? [Vous pouvez sélectionner plus d'une réponse]  1, Information  2, Medicaments  3, Conseils/thérapie  4, Soutien par les pairs  5, Autre (Veuillez spécifier) |
| 27** | Who was the person that recommended you/referred you to this mental health service provider?  1, Family member  2, Friend, or colleague  3, Mental health service provider  (i.e. doctor, psychiatrist, psychologist,  social worker, counsellor)  4, Other professional  (i.e. teacher, youth support worker)  5, Spiritual/religious guide or traditional healer  6, Myself  7, Other (Please specify) | Qui vous a recommandé ce/cette professionnel-le de la santé mentale ou qui vous a aiguillé-e vers lui/elle?  1, Un membre de votre famille  2, Un ami ou un collègue  3, Un-e professionnel-le de la santé mentale  (ex: médecin, psychiatre, psychologue, travailleur/se social-e, thérapeute)  4, Un-e autre professionnel-le  (ex: enseignant-e, intervenant-e jeunesse)  5, Un-e chef-fe spirituel-le ou un-e guérisseur/se traditionnel-le  6, Moi-Même  7, Autre (Veuillez spécifier) |
| 28 | Please describe the steps you went through to access this mental health service provider: | Si possible, svp nous décrire les étapes que vous avez suivies pour avoir accès à ce/cette professionnel-le de santé mentale |
| 29 | How did you contact this mental health service provider?  1, By phone (call/text)  2, By e-mail  3, Through an online system  4, In-Person  5, Other (Please specify) | Comment avez-vous contacté ce/cette professionnel-le de santé mentale?  1, Par téléphone  2, Par courriel  3, Via une plateforme en ligne  4, En personne  5, Autre (Veuillez spécifier) |
| 30 | Who filled out the initial referral forms for this mental health service?  1, Family member  2, Friend or colleague  3, Mental health service provider  (i.e. doctor, psychiatrist, psychologist,  social worker, counsellor)  4, Other professional  (i.e. teacher, youth support worker)  5, Spiritual/religious guide or traditional healer  6, Myself  7, Other (Please specify) | Qui a remplit les formulaires de référence pour ce service de santé mentale?  1, Un membre de votre famille  2, Un ami ou un collègue  3, Un-e professionnel-le de la santé mentale  (ex: médecin, psychiatre, psychologue, travailleur/se social-e, thérapeute)  4, Un-e autre professionnel-le  (ex: enseignant-e, intervenant-e jeunesse)  5, Un-e chef-fe spirituel-le ou un-e guérisseur/se traditionnel-le  6, Moi-Même  7, Autre (Veuillez spécifier) |
| 31 | Approximately how long were you on the waitlist for?  1, Less than a week  2, One to three weeks  3, One to three month(s)  4, Four to six months  5, Seven to nine months  6, Ten to twelve months  7, More than a year | Pendant environ combien de temps êtes-vous resté-e sur la liste d'attente?  1, Moins d'une semaine  2, Entre une semaine et trois semaines  3, Entre un mois et trois mois  4, Entre quatre mois et six mois  5, Entre sept mois et neuf mois  6, Entre dix mois et douze mois  7, Plus d'un an |
| 32 | Please elaborate on any information that could help us understand how you accessed this mental health service provider | Veuillez svp rajouter des explications qui pourraient nous aider à mieux comprendre comment vous avez eu accès à ce/cette professionnel-le de santé mentale |
| 33 | Which of the following best describes the MAIN obstacle you experienced when trying to receive mental health services from this mental health service provider?  1, Personal stigma  (i.e. worries/anxiety about judgement from others,  being a burden, the help-seeking process)  2, Social stigma and discrimination  (i.e. racism, sexism, homophobia, transphobia,  classism, judgement/insensitivity from others)  3, Confidentiality issues  (i.e. concerns about others finding out/  service provider telling others)  4, Lack of knowledge  (i.e. about mental health and mental illness, about mental health services available)  5, Concerns about the service provider  (i.e. worried they wouldn't help/meet my needs, uncomfortable talking to them)  6, Wait times  (i.e. long wait lists, service provider was busy)  7, Accessibility issues  (i.e. service location, cost,  difficulty getting in contact with service providers)  8, Communication issues  (i.e. language barriers)  9, Preferred help from other sources  (i.e. myself, friends, family, religious leaders)  10, Disability  (e.g. related to physical mobility,  visual impairment, auditory and speech impairment)  11, Scheduling conflicts  12, Other (Please specify)  13, I'm not sure  14, I did not experience any obstacles | Laquelle des options ci-dessous décrit le mieux le PRINCIPAL obstacle que vous avez rencontré lorsque vous cherchiez à recevoir des services de santé mentale de la part de ce/cette professionnel-le de santé mentale?  1, Stigmatisation personnelle  (ex: inquiétude/anxiété liée à la possibilité d'être jugé-e ou de représenter un fardeau pour les autres ou par rapport au processus de demande d'aide)  2, Stigmatisation et discrimination sociales  (ex: racisme, sexisme, homophobie, transphobie, préjugés de classe, jugement/manque de sensibilité de la part d'autrui)  3, Inquiétudes liées à la confidentialité  (ex: soucis que d'autres personnes l'apprennent ou que le/la professionnel-le en parle à d'autres)  4, Connaissances insuffisantes  (ex: à propos de la santé mentale et de la maladie mentale, à propos des services disponibles en santé mentale)  5, Inquiétudes liées au/à la professionnel-le  (ex: inquiétude qu'il/elle ne pourrait pas m'aider/répondre à mes besoins, inconfort dans le fait de parler avec lui/elle)  6, Temps d'attente  (ex: longues listes d'attente, manque de disponibilité du/de la professionnel-le)  7, Accessibilité  (ex: emplacement des services, coût, difficulté à rejoindre le/la professionnel-le)  8, Communication  (ex: barrières linguistiques)  9, Préféré trouver de l'aide ailleurs  (ex: de moi-même, de mes amis, de ma famille, d'un-e chef-fe religieux/se)  10, Incapacité  (ex: liée à la mobilité physique, déficience visuelle ou auditive, troubles de parole et de langage)  11, Conflits d'horaire  12, Autre (Veuillez spécifier)  13, Je ne sais pas  14, Je n'ai pas rencontré d'obstacles |
| 34 | Based on your experience, how would you rate the process of referral to this mental health service provider?  1, Very unsatisfactory  2, Unsatisfactory  3, Neutral  4, Satisfactory  5, Very satisfactory | Selon votre expérience, comment évalueriez-vous le processus de référence vers ce/cette professionnel-le de santé mentale?  1, Très insatisfaisant  2, Plutôt insatisfaisant  3, Neutre  4, Plutôt satisfaisant  5, Très satisfaisant |
| 35 | Did this mental health service provider refer/recommend you to another service provider?  Yes  No | Est-ce que ce/cette professionnel-le de santé mentale vous a aiguillé-e ou recommandé un-e autre professionnel-le?  Oui  Non |
| 36 | How was that referral made?  1, By phone (call/text)  2, By e-mail  3, Through an online system  4, In-Person  5, Other (Please specify) | Par quel moyen la référence a-t-elle été acheminée?  1, Par téléphone  2, Par courriel  3, Via une plateforme en ligne  4, En personne  5, Autre (Veuillez spécifier) |
| 37 | Which service provider did they refer you to?  1, Doctor  2, Counsellor  3, Psychologist  4, Psychiatrist  5, Other mental health service provider  (e.g. nurse, social worker...)  6, Peer  7, I'm not sure  8, Other (Please specify) | Vers quel-le professionnel-le vous a-t-il(elle) aiguillé-e?  1, Médecin  2, Thérapeute  3, Psychologue  4, Psychiatre  5, Autre professionnel-le de la santé mentale (ex: infirmière, travailleur/se social-e...)  6, Pair-aidant  7, Je ne sais pas  8, Autre (Veuillez spécifier) |
| 38 | Where was this mental health service provider located?  1, Public Clinic (e.g. local health centre, hospital)  2, Private Clinic (e.g. psychologist, psychotherapist)  3, Emergency Room  4, School Counselling or Health Services  5, Telephone Help Line  6, Community Organization  7, Online  8, Other (Please specify) | Où était situé-e ce/cette professionnel-le de santé mentale?  1, Clinique Publique  2, Clinique Privée (e.g. psychologue, psychothérapeute)  3, Urgence  4, Services de santé de l'école  5, Service d'aide téléphonique  6, Organisme communautaire spécialisé en santé mentale  7, Services d'aide en ligne  8, Autre (Veuillez spécifier) |
| 39 | Did you receive mental health services from the mental health service provider you were referred to?  Yes  No | Avez-vous reçu des services de santé mentale de la part du/de la professionnel-le de santé mentale à qui vous avez été aiguillé-e?  Oui  Non |
| 40 | Approximately how long did it take you to get an appointment with the mental health service provider you were referred to?  1, Less than a week  2, One to three weeks  3, One to three month(s)  4, Four to six months  5, Seven to nine months  6, Ten to twelve months  7, More than a year | Environ combien de temps cela vous a-t-il pris avant d'avoir un rendez-vous avec le/la professionnel-le à qui vous avez été aiguillé-e?  1, Moins d'une semaine  2, Entre une semaine et trois semaines  3, Entre un mois et trois mois  4, Entre quatre mois et six mois  5, Entre sept mois et neuf mois  6, Entre dix mois et douze mois  7, Plus d'un an |
| 41 | Which type of service(s) did you receive from this mental health service provider? [Please select all that apply]  1, Information  2, Medication  3, Counselling/Therapy  4, Peer support  5, Other (Please specify) | Quel(s) type(s) de service(s) avez-vous reçu(s) de la part de ce fournisseur de services en santé mentale? [Vous pouvez sélectionner plus d'une réponse]  1, Information  2, Medicaments  3, Conseils/thérapie  4, Soutien par les pairs  5, Autre (Veuillez spécifier) |
| 42 | Which of the following best describes the MAIN obstacle you experienced when trying to receive mental health services from the mental health service provider you were referred to?  1, Personal stigma  (i.e. worries/anxiety about judgement from others,  being a burden, the help-seeking process)  2, Social stigma and discrimination  (i.e. racism, sexism, homophobia, transphobia,  classism, judgement/insensitivity from others)  3, Confidentiality issues  (i.e. concerns about others finding out/  service provider telling others)  4, Lack of knowledge (i.e. about mental health and mental illness, about mental health services available)  5, Concerns about the service provider  (i.e. worried they wouldn't help/meet my needs,  uncomfortable talking to them)  6, Wait times  (i.e. long wait lists, service provider was busy)  7, Accessibility issues  (i.e. service location, cost,  difficulty getting in contact with service providers)  8, Communication issues  (i.e. language barriers)  9, Preferred help from other sources  (i.e. myself, friends, family, religious leaders)  10, Disability  (e.g. related to physical mobility,  visual impairment, auditory and speech impairment)  11, Scheduling conflicts  12, Other (Please specify)  13, I'm not sure  14, I did not experience any obstacles | Laquelle des options ci-dessous décrit le mieux le PRINCIPAL obstacle que vous avez rencontré lorsque vous cherchiez à recevoir des services de santé mentale de la part du/de la professionnel-le de santé mentale à qui vous avez été aiguillé-e?  1, Stigmatisation personnelle  (ex: inquiétude/anxiété liée à la possibilité d'être jugé-e ou de représenter un fardeau pour les autres ou par rapport au processus de demande d'aide)  2, Stigmatisation et discrimination sociales  (ex: racisme, sexisme, homophobie, transphobie, préjugés de classe, jugement/manque de sensibilité de la part d'autrui)  3, Inquiétudes liées à la confidentialité  (ex: soucis que d'autres personnes l'apprennent ou que le/la professionnel-le en parle à d'autres)  4, Connaissances insuffisantes  (ex: à propos de la santé mentale et de la maladie mentale, à propos des services disponibles en santé mentale)  5, Inquiétudes liées au/à la professionnel-le  (ex: inquiétude qu'il/elle ne pourrait pas m'aider/répondre à mes besoins, inconfort dans le fait de parler avec lui/elle)  6, Temps d'attente  (ex: longues listes d'attente, manque de disponibilité du/de la professionnel-le)  7, Accessibilité  (ex: emplacement des services, coût, difficulté à rejoindre le/la professionnel-le)  8, Communication  (ex: barrières linguistiques)  9, Préféré trouver de l'aide ailleurs  (ex: de moi-même, de mes amis, de ma famille, d'un-e chef-fe religieux/se)  10, Incapacité  (ex: liée à la mobilité physique, déficience visuelle ou auditive, troubles de parole et de langage)  11, Conflits d'horaire  12, Autre (Veuillez spécifier)  13, Je ne sais pas  14, Je n'ai pas rencontré d'obstacles |
| 43 | Based on your experience, how would you rate the process of referral to this mental health service provider?  1, Very unsatisfactory  2, Unsatisfactory  3, Neutral  4, Satisfactory  5, Very satisfactory | Selon votre expérience, comment évalueriez-vous le processus de référence vers ce/cette professionnel-le de santé mentale?  1, Très insatisfaisant  2, Plutôt insatisfaisant  3, Neutre  4, Plutôt satisfaisant  5, Très satisfaisant |
| 44 | Approximately how many mental health service providers in total did you contact before connecting to one that addressed your needs and concerns?  1  2  3  4  5  6+ | Environ combien de professionnel-le-s de santé mentale avez-vous contacté-e-s avant de rencontrer un-e qui a répondu à vos besoins et à vos préoccupations?  1  2  3  4  5  6+ |
| 45 | Overall, approximately how long did it take for you to  connect to a mental health service provider  that addressed your needs and concerns?  1, Less than a week  2, One to three weeks  3, One to three month(s)  4, Four to six months  5, Seven to nine months  6, Ten to twelve months  7, More than a year | Environ combien de temps cela vous a-t-il pris avant de joindre un-e professionnel-le de la santé mentale qui a répondu à vos besoins et à vos préoccupations?  1, Moins d'une semaine  2, Entre une semaine et trois semaines  3, Entre un mois et trois mois  4, Entre quatre mois et six mois  5, Entre sept mois et neuf mois  6, Entre dix mois et douze mois  7, Plus d'un an |
| 46 | Based on your experience, how would you rate the overall process of referral to the mental health service provider that addressed your needs and concerns?  1, Very unsatisfactory  2, Unsatisfactory  3, Neutral  4, Satisfactory  5, Very satisfactory | En général, selon votre expérience, comment évalueriez-vous le processus de référence vers le/la professionnel-le de santé mentale qui a répondu à vos besoins et à vos préoccupations?  1, Très insatisfaisant  2, Plutôt insatisfaisant  3, Neutre  4, Plutôt satisfaisant  5, Très satisfaisant |
| ONLINE REFERRAL TOOL | | |
| 47 | Imagine that there was an online tool that allowed youth to refer themselves to mental health services. Youth would be asked to provide basic information about themselves and the concern that they are seeking help for. They would choose the way that they would like to be contacted by the clinician (for example by phone, email, text messaging, live chat, or video-conferencing). | Imaginez qu'il existe un outil en ligne permettant aux jeunes de s'autoréférer aux services de santé mentale. On inviterait les jeunes à fournir quelques informations sur eux-mêmes et sur les préoccupations pour lesquelles ils/elles demandent de l’aide. Ils/elles choisiraient la manière dont ils/elles aimeraient être contacté-e-s par l'intervenant-e (ex: par téléphone, par courriel, par texto, par clavardage ou par vidéoconférence) |
| 48 | In which ways do you think this tool could be HELPFUL for improving the referral experience and process for a young person? | De quelles manières pensez-vous que cet outil pourrait AIDER à améliorer le processus de référence et l'expérience des jeunes par rapport aux références? |
| 49 | In which ways do you think this tool could be LESS HELPFUL for improving the referral experience and process for a young person? | De quelles manières pensez-vous que cet outil pourrait NE PAS AIDER à améliorer le processus de référence et l'expérience des jeunes par rapport aux référence? |
| 50 | If an online self-referral tool for mental health services was available, how likely would you be to use it if you needed mental health support?  1, Very unlikely  2, Unlikely  3, Neutral  4, Likely  5, Very Likely | S'il existait un outil d'auto-référence en ligne pour des services de santé mentale, dans quelle mesure serait-il probable que vous l'utilisiez si vous aviez besoin d'un soutien de santé mentale?  1, Très peu probable  2, Peu probable  3, Neutre  4, Probable  5, Très probable |
| 51 | Please provide two key recommendations regarding this tool | Veuillez formuler deux recommandations clé pour cet outil |
| 52 | I agree that the information I have provided in this survey can be used by the research team to carry out other research projects in the field of mental health  1, Yes  2, No | Je suis d’accord que l’information que j’ai fournie dans ce sondage puisse être utilisée par l’équipe de recherche dans d’autres projets de recherche dans le domaine de la santé mentale  1, Oui  2, Non |
|  | END OF SURVEY | |
|  | This is the end of the survey.  By clicking “✓” you are providing your consent for the  information you provided to be saved  and collected by the researchers.  Once you click submit you will not be able to withdraw your  data as the survey is anonymous and the researchers will not  be able to identify which data is yours.  Data will be stored for 10 years in  accordance with the institution's ethics policies. | C'est la fin du sondage.  En cliquant sur "✓" vous consentez à ce que l'information que vous avez fournie soit enregistrée et collectée par les chercheurs.  Une fois que vous aurez cliqué sur "submit" vous ne pourrez plus retirer vos données puisque le sondage est anonyme et que les chercheurs ne pourront plus identifier vos réponses.  Les données seront conservées pendant 10 ans, conformément aux politiques institutionnelles en matière d'éthique. |

1. Ethnicity question and responses were adapted using information from the following documents: 1) Statistics Canada Census of Population 2016, Appendix 5.1 Ethnic Origins disseminated from 2016, 2011, and 2006. Retrieved from: <https://www12.statcan.gc.ca/census-recensement/2016/ref/dict/app-ann/a5_1-eng.cfm>, 2) Archived Census 2A-L - 2016. Retrieved from: <http://www23.statcan.gc.ca/imdb/p3Instr.pl?Function=getInstrumentList&Item_Id=295122&UL=1V&>, 3) Ethnic Origin Reference Guide, Census of Population, 2016. Retrieved from: <https://www12.statcan.gc.ca/census-recensement/2016/ref/guides/008/98-500-x2016008-eng.cfm>,

   & 4) Data Tables 2016 Census: Ethnic Origins. Retrieved from: <https://www12.statcan.gc.ca/census-recensement/2016/dp-pd/dt-td/Av-eng.cfm?LANG=E&APATH=3&DETAIL=0&DIM=1&FL=A&FREE=0&GC=0&GID=0&GK=0&GRP=1&PID=112450&PRID=10&PTYPE=109445&S=0&SHOWALL=0&SUB=0&Temporal=2017&THEME=120&VID=29591&VNAMEE=&VNAMEF=> [↑](#footnote-ref-1)
