# Supplementary material for: Youth Experiences With Referrals to Mental Health Services in Canada: Protocol for a Web-Based Cross-Sectional Survey Study
Source: JMIR Res Protoc. 2020 Mar 24;9(3):e16945. doi: 10.2196/16945 (PMC7139421; doi:10.2196/16945)
Supplement: Multimedia Appendix 3 [file resprot_v9i3e16945_app3.pdf]

Mitacs

November 15, 2017

Application Ref. IT10339

RE: Mitacs Accelerate Proposal

**Project Title:**	**Development, Implementation and Evaluation of PRISM-ACCESS Open Minds: Pathway for Rapid, Internet-based, Self-referral to Mental health services for youth**
**Internship Supervisor(s):**	**Shalini Lal**
**Intern(s):**	**, TBD**
**Department:**	**Faculté de médecine, École de réadaptation**
**Institution:**	**Université de Montréal**
**Partner Organization:**	**Graham Boeckh Foundation**

Dear Dr. Shalini Lal,

Your application to Mitacs Accelerate has successfully passed research review. Your **research project** has been approved for a total grant of $80,000.00 that will be delivered through **eligible internships**. Your internship should not start prior to confirmation of eligibility as outlined below:

1. Mitacs must receive partner funds before each internship start date to pay the intern(s) on time;
2. Project leads must confirm intern name(s) and start date(s). No costs for an internship can be incurred prior to the research project approval and the receipt of partner funds for the internship;
3. To identify a new intern, the Intern Profile form (including CV and memorandum) must be submitted to Mitacs before the applicable internship's start date and obtain Mitacs approval;
4. Project leads must obtain the following certification(s) or approval(s) before the project begins: University Research Ethics Board approval.

All awards are subject to ongoing program eligibility, continuation of funding by the partner organization(s), and continuation of funding by our government partners. Mitacs-Accelerate gratefully acknowledges the financial support of the Government of Canada and the Government of Québec through the Ministère de l'Économie, de la Science et de l'Innovation (MESI).

Welcome to Mitacs Accelerate. With this program, interns can apply their specialized expertise while companies gain a competitive advantage. We look forward to the success of your project.

If you have any questions, please contact Rebekah Thompson, Spécialiste de la Gestion des Subventions/Grants Specialist, at rthompson@mitacs.ca.

Sincerely,

Olga Stachova
Chief Operating Officer

Enclosures:

- Appendix A: Referee comments
- Appendix B: Terms and Conditions of the Award
- Attachment: Intern Onboarding Information

Appendix A: Referee Comments

Your application has been reviewed by three external referees. Altogether, they are supportive of the internship proposal and have found the project interesting with a clear research orientation. The proposal provides sufficient details on the rationale, motivations, objectives and proposed solutions. We invite you to take into account the suggestions provided by Reviewer 1 in question #4 and Reviewer 2 in questions # 4 and 6 that might be helpful in further enhancing the methodology.

Please find below the full reviewer reports for your reference. We wish the applicants good luck in their endeavor.

Reviewer 1

1) Mitacs Accelerate supports research-based internships. Does the proposed project qualify as research in its discipline?
Tout à fait. Le programme de recherche proposé par la candidate est tout à fait justifié sur le plan scientifique et sur le plan socio-sanitaire. Les internes seront accueillis dans un milieu riche et pourront acquérir des habiletés de recherche de qualité. Leurs productions seront utiles pour faire avancer les connaissances dans le domaine de l'accessibilité aux soins de santé mentale.

2) Is the project appropriate for the academic degree level of the intern?
Le niveau universitaire des internes n'est pas indiqué. A mon avis, le niveau exige, au minimum, une formation théorique des études supérieures, idéalement une maîtrise. Un niveau plus élevé serait approprié afin de favoriser l'autonomie et respecter l'échéancier.

3) Are the objectives clear?
Tout à fait. Les objectifs sont très clairs et permettront aux internes de bien cibler leur contribution dans un ensemble plus grand et très cohérent.

4) Is the methodology appropriate to achieve the objectives?
Tout à fait. Le projet est très cohérent et l'approche méthodologique, semble suffisamment traditionnelle afin que les internes puissent s'y retrouver. Ma seule réserve concerne le recrutement pour l'enquête en-ligne : l'expression « implement survey » reste assez nébuleuse étant donné que cette population peut être difficile à rejoindre. Je me demande, et c'est vraiment une réflexion, si la stratégie des entrevues en profondeur n'est pas plus faisable et informative.

5) Is the timeline realistic?
L'échéancier est serré et faisable pour deux des internes. Je reste plus inquiète pour l'enquête qui doit passer au Comité d'éthique à la recherche et qui doit compter sur la participation de jeunes.

6) Other comments and suggestions for the applicants. For example, is there prior work that should be considered by the researchers?
Ma principale suggestion a été écrite à la question 4.

Reviewer 2

1) Mitacs Accelerate supports research-based internships. Does the proposed project qualify as research in its discipline?
The project seems appropriate and does qualify as research in the discipline identified.

2) Is the project appropriate for the academic degree level of the intern?
This is unclear as all three interns are TBD, so I have no sense of their academic level or research experience based on what is provided by the applicant. The three projects are not particularly complex but one does need to know how to do a good literature search and review and understand survey methodology. Strong social or health

sciences studied, even at upper undergrad levels, should be able to do the tasks as outlined.

3) Are the objectives clear?
Yes.

4) Is the methodology appropriate to achieve the objectives?
Yes.

5) Is the timeline realistic?
Yes and may be generous. The tasks the interns will do seem to be preliminary to the main RTC but that is appropriate. The survey would be an update of previous research by the principal investigator.

6) Other comments and suggestions for the applicants. For example, is there prior work that should be considered by the researchers?
The reference list is comprehensive and relevant and I am unaware of any major work that has not been cited.

Reviewer 3

1) Mitacs Accelerate supports research-based internships. Does the proposed project qualify as research in its discipline?
Yes this project qualifies as research.

2) Is the project appropriate for the academic degree level of the intern?
Yes the tasks identified are appropriate for an intern.

3) Are the objectives clear?
The objectives are clear and well thought out.

4) Is the methodology appropriate to achieve the objectives?
This is an interesting and innovative project. The goals of the project are reasonable, feasible and timely. The projected roles for the interns fit the goals of the project. Self referral is an important avenue for youth to seek mental health treatment. But it makes two important assumptions, that the investigator should recognize. First is that youth have a self awareness sufficient to recognize that mental health treatment might be useful to them. Second, that the mental health treatment would actually be useful to them. It would seem a bit naive to assume that youth with all types of mental health challenges are equally likely to be able and willing to self refer. Some attention to individual difference in mental health needs and strengths would enhance this proposal dramatically.

5) Is the timeline realistic?
The timeline is realistic.

6) Other comments and suggestions for the applicants. For example, is there prior work that should be considered by the researchers?
With regard to the first intern, the investigator describes the methodology as an RCT however, randomization of six sites into three groups is fraught with methodological shortcomings and likely make the interpretation of the finding using the traditional RCT frame potentially misleading.
Regardless, the project has few other reasonable methodological options given the nature of this intervention. I would just suggest that both caution against either over or under-interpretation of the findings is potentially limiting. RCT designs allow a yes/no dichotomous judgment-the intervention worked or it didn't. That is great for establishing an evidence-based practice. However, it can be very limiting when understanding the nuances of a new technology.
For the second intern, I would suggest that the environmental scan of social media is not likely to benefit dramatically from a scientific literature review. Social media is evolving at a far faster cycle than traditional journal publications so rapid response surveys of youth are far more likely to identify useful social media approaches and working with common search engines to increase visibility are likely more fruitful endeavors that a review of the extant scientific literature.
Finally, for the third intern, it is unclear who the sample would be for the youth survey. I would suggest it should

be a broad sample and not simply a convenience sample of you who self referred. Of course, the most interesting questions involve youth who might decide they did not wish to self-refer rather than understanding those who did.

Appendix B: Mitacs Accelerate - Terms and Conditions of the Award

Please read all conditions carefully. Any questions or requests for clarification should be directed to your Grant Management Specialist listed in the letter above.

1.General guidelines:

1.1.The average amount of partner interaction for each four-month internship across the project should be 50% (or minimum of 25% with sufficient justification in original application).

1.2.Interns may complete up to 2 internship segments (4-6 months each) during a Master's degree, and up to 6 during a PhD and Post-doctoral Fellows may complete a maximum of 6 internship segments.

1.3.The start date of each internship cannot predate the research approval of the project and the receipt of partner funds for the internship.

1.4.Awarded funds must be allocated according to the internship period(s) and stipend/salary allocations, which will be outlined on the Mitacs Award Letter.

1.5.Please report any substantial change (internship dates, project personnel, partner organizations, etc.) concerning your project immediately to your Mitacs Grant Management Specialist contact.

2.Funding:

2.1.Contribution funds from the partner organization must be paid to Mitacs prior to the internship start date to facilitate efficient payment process for interns.

2.2.Funding will not be released for un-named interns.

2.3.The stipend portion of the award must be spent as outlined in the award letter. No extensions can be granted on stipend amounts.

2.4.The research grant portion of the award must be spent by end date of the award. Any remaining research funds may be used by the academic supervisor to supplement the intern's stipend at the Academic Supervisor's discretion. Any funds not spent by this date must be returned to Mitacs unless an extension of the award is granted by Mitacs. To obtain an extension, the supervisor needs to contact Mitacs before the end date of the award, with justification of such a request.

2.5.In-kind contributions cannot be used towards matching Mitacs funds.

2.6.Inter-university transfers are not permitted.

2.7.To ensure that your project complies with the Mitacs university agreement and federal granting council policies, you must provide Mitacs with copies of all contracts that govern the research being performed using Mitacs funds.

3.Reporting

3.1.Each intern **must** receive a minimum $10,000 stipend/salary for each internship unit.

3.2.All stipend/salary funds must be paid over a period of <u>no less than 4 months and no greater than 6 months</u> per internship unit.

4.Research Funds:

4.1.The remaining funding may be used to cover expenses in support of the internship research and its related costs, as directed by Tri-Council. A detailed description of eligible expenses is available on the NSERC website at the following link: http://www.nserc-crsng.gc.ca/Professors-Professeurs/FinancialAdminGuide-GuideAdminFinancier/FundsUse-UtilisationSubventions_eng.asp

4.2.Relevant research expenses incurred outside of the indicated funding period may be approved by Mitacs on a case-by-case basis.

5.Reporting:

5.1.If the award straddles fiscal years, the University will provide a separate Form 300 (statement of account) for each account by **May 31st** for each fiscal year until the account reaches a zero balance. The University will provide a Final Form 300 for each account **within 30 days of the end date of this award.** The Academic Supervisor and Finance Officer must sign the Form 300s and forward to Mitacs once finalized. **Please include the Application Reference number and Project Title on the Form 300 and submit to** form300@mitacs.ca

5.2.Projects involving more than 50 internship units and spanning longer than 2 years are required to submit a progress report to Mitacs at the mid-point of the project. Please designate one academic supervisor to be the coordinator who is responsible for submitting the report to Mitacs. Failure to submit a satisfactory report may result in delay or cancellation of subsequent funding.

5.3.All parties are required to provide Mitacs with a final report and completed exit surveys at the end of the Mitacs Accelerate internship. Please find the links below:
Accelerate Final Report:
http://www.mitacs.ca/sites/default/files/uploads/page/mitacs_accelerate_final_report_mar2013.zip
Accelerate Intern Exit Survey:
http://sondages.outsidesoft.com/Engine/Default.aspx?surveyID=9eff01b6-316a-49c9-b257-83afff51e497&lang=EN
Accelerate Professor Exit Survey:
http://sondages.outsidesoft.com/Engine/Default.aspx?surveyID=023afce3-c581-4c97-9294-6e8f5e25b1ef&lang=EN
Accelerate Partner Exit Survey:
http://sondages.outsidesoft.com/Engine/Default.aspx?surveyID=f413d47d-b4f3-49a4-be2c-5e0a947bcafb&lang=EN

6.Acknowledgement of Mitacs:

6.1.All Mitacs-sponsored results must explicitly credit Mitacs. This includes work by professors, post-docs, interns, and research associates. Mitacs funding can be acknowledged in publications using the following statement: ''This work was supported by Mitacs through the Mitacs Accelerate Program.'' All networking activities initiated by your project for which Mitacs funds are used must also credit Mitacs. For advertising purposes, the Mitacs logo is available in several formats for download from the website at www.mitacs.ca
